# Clay Minerals as Bioink Ingredients for 3D Printing and 3D Bioprinting: Application in Tissue Engineering and Regenerative Medicine

**DOI:** 10.3390/pharmaceutics13111806

**Published:** 2021-10-28

**Authors:** Fátima García-Villén, Sandra Ruiz-Alonso, Markel Lafuente-Merchan, Idoia Gallego, Myriam Sainz-Ramos, Laura Saenz-del-Burgo, Jose Luis Pedraz

**Affiliations:** 1 NanoBioCel Group, Laboratory of Pharmaceutics, School of Pharmacy, University of the Basque Country UPV/EHU, 01006 Vitoria-Gasteiz, Spain; sandra.ruiz@ehu.eus (S.R.-A.); mlafuente004@ikasle.ehu.eus (M.L.-M.); idoia.gallego@ehu.eus (I.G.); miriam.sainz@ehu.eus (M.S.-R.); laura.saenzdelburgo@ehu.eus (L.S.-d.-B.); 2 Biomedical Research Networking Center in Bioengineering, Biomaterials and Nanomedicine (CIBER-BBN), 01006 Vitoria-Gasteiz, Spain; 3 Bioaraba, NanoBioCel Resarch Group, 01009 Vitoria-Gasteiz, Spain

**Keywords:** clay, nanosilicate, phyllosilicate, tissue engineering, regenerative medicine, 3D printing, 3D bioprinting, bioink

## Abstract

The adaptation and progress of 3D printing technology toward 3D bioprinting (specifically adapted to biomedical purposes) has opened the door to a world of new opportunities and possibilities in tissue engineering and regenerative medicine. In this regard, 3D bioprinting allows for the production of tailor-made constructs and organs as well as the production of custom implants and medical devices. As it is a growing field of study, currently, the attention is heeded on the optimization and improvement of the mechanical and biological properties of the so-called bioinks/biomaterial inks. One of the strategies proposed is the use of inorganic ingredients (clays, hydroxyapatite, graphene, carbon nanotubes and other silicate nanoparticles). Clays have proven to be useful as rheological and mechanical reinforcement in a wide range of fields, from the building industry to pharmacy. Moreover, they are naturally occurring materials with recognized biocompatibility and bioactivity, revealing them as optimal candidates for this cutting-edge technology. This review deals with the use of clays (both natural and synthetic) for tissue engineering and regenerative medicine through 3D printing and bioprinting. Despite the limited number of studies, it is possible to conclude that clays play a fundamental role in the formulation and optimization of bioinks and biomaterial inks since they are able to improve their rheology and mechanical properties, thus improving printability and construct resistance. Additionally, they have also proven to be exceptionally functional ingredients (enhancing cellular proliferation, adhesion, differentiation and alignment), controlling biodegradation and carrying/releasing actives with tissue regeneration therapeutic activities.

## 1. Introduction

Three-dimensional printing (3D printing) is a relatively recent technique used to model and fabricate three-dimensional constructs with a layer-by-layer procedure. In this technique, computer or software-aided design (CAD) is used to create and generate a g-code that will be read by the 3D printer to fabricate the desired item. Currently, there are several 3D printing technologies available, depending on the nature of the materials used for the printing process and the final scope of the printed construct itself. Since its appearance, 3D printing has influenced a significant number of areas, from construction, mechanics, industry or art to medicine and healthcare. Its versatility and potential are especially highlighted when it comes to the biomedical field, area in which it acquires the denomination “3D bioprinting” (3DBP) or “bioprinting” to clearly differentiate it from the rest of the 3D printing applications (Table 1).

Bioprinting is a growing and promising technology that allows for the fabrication of new dosage forms, tailor-made devices and implants, tailor-made scaffolds for tissue engineering (TE), and many other individualized therapy options [1]. Until the appearance of 3DBP, the major part of the TE approaches was based and limited to two-dimensional cell sheets. Currently, 3DBP is able to provide cell-laden three-dimensional structures with extracellular matrix (ECM) ingredients possessing the right shape and size to be directly implanted in a certain patient. Moreover, 3DBP offers the possibility to reproduce complex tissue-engineered constructs.

“Bioink” (BI) is the term used to refer to a cell-laden, biocompatible material intended to be three-dimensionally bioprinted [2]. The concept of BI is, in fact, what defines the boundary between 3DBP and conventional 3DP (Table 1) [1,3]. Most of the time, the BI formulations are hydrogel-based since they are formed by water-rich materials that more closely reproduce the extracellular matrix (ECM) environment. On the contrary, if cells are not present in the material to be printed, it can be defined as a “biomaterial ink” (BMI) [2,4]. Even if the BMI is printed and cells are subsequently seeded over the printed construct, this formulation does not qualify as BI, as suitable pointed out by Groll and co-workers [2]. The use of biocompatible and biodegradable ingredients, together with the inclusion of cells within the so-called BI, make it possible to print customized structures and/or tissues with minimal healing time as well as minimal chances of implant rejection and other immune responses. At present, 3DBP has enabled the in vitro production of complex tissues such as skin, cartilage, bone, lung or heart. 3DBP can be defined as a “super multi-discipline technology involving tissue biology, cell biology, computer technology, materials science and medical sciences” [5].

Biocompatible polymers (both natural and synthetic) are the main ingredient of BIs and BMIs. Among today’s most used natural BI ingredients, alginate, gelatin, collagen (type I), chitosan, silk fibroin, albumin, agarose, thrombin, fibrinogen, hyaluronic acid, chitosan, chitin and cellulose [1,6] can be highlighted. These ingredients are able to form hydrogels (by different mechanisms), thus retaining high amounts of water and offering remarkable porosity. These aqueous and porous networks enable the permeability and diffusion of nutrients and gasses, thus emulating a favorable micro-environment for cell growth. Nonetheless, the major part of the aforementioned natural ingredients possesses poor mechanical properties, shape and time-stability, which are crucial features for bioprinting. For these reasons, synthetic, biocompatible ingredients such as gelatin methacryloyl, pluronic, polyethylene glycol, polyglycolic acid, poly(lactic acid), polycaprolactone or polylactic-co-glycolic are used and often combined with natural ingredients in order to reach a correct balance between biocompatibility, biodegradability, functionality and proper mechanical properties of the BI or BMI [1]. Regarding the improvement of BI mechanical properties, some inorganic ingredients, particularly those with well-known biocompatibility such as hydroxyapatite, graphene, carbon nanotubes, clay minerals, zeolites and other silicate nanoparticles, are currently in the spotlight [1,3,7,8,9,10]. The present review specifically focuses on the current uses of clay minerals for 3DBP, aiming to provide a comprehensive overview of the potential of these minerals as ingredients of BIs and BMIs. The following section will be specifically dedicated to describing these inorganic ingredients, including an overview of their most characteristic physicochemical properties.

### 1.1. Clay Minerals

**Figure 1:**
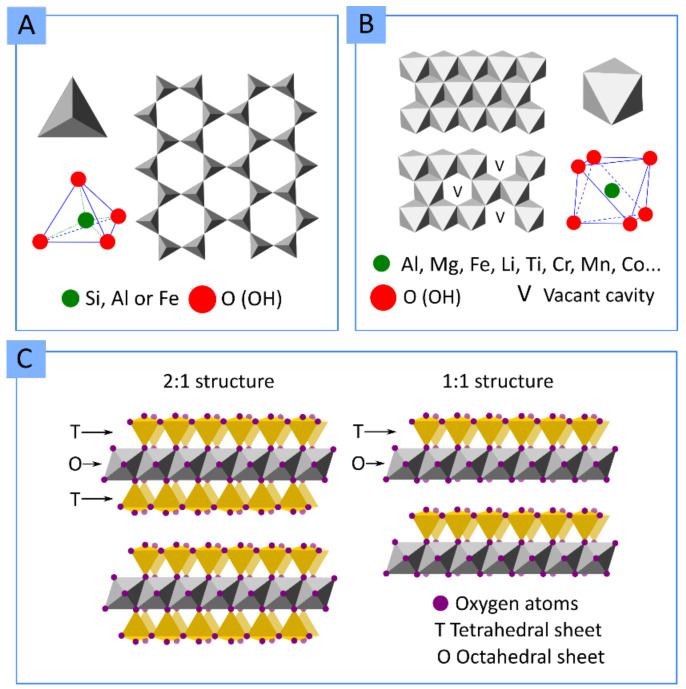
Schematic representation of (**A**) basic phyllosilicate tetrahedron and spatial disposition of tetrahedral sheets and (**B**) octahedron and spatial disposition of octahedral sheets. (**C**) Phyllosilicates layer structures 2:1 (T:O:T) and 1:1 (T:O).

The term “clay mineral” refers to a group of minerals (natural or synthetic) grouped as “phyllosilicates”. Chemically speaking, phyllosilicates are hydrated aluminosilicate minerals composed of aluminum and silicon oxides that also contain a high amount of cations such as Mg, K, Ca, Na and Fe. Structurally speaking, phyllosilicates are formed by continuous, stacked, tetrahedral and octahedral sheets. Each tetrahedron consists of Si^4+^ cations coordinated to four oxygen atoms orientated in an infinite two-dimensional hexagonal mesh pattern (Figure 1A). The octahedral stratum is formed by the coordination of metal cations (mainly Mg, Al, Fe, Li, etc.) with oxygen atoms, OH^−^ or F^−^ of the tetrahedral sheet. Each octahedron share edges with one another to form a continuous sheet with hexagonal or pseudo-hexagonal symmetry (Figure 1B). The tetrahedral and octahedral sheets are associated with the apical oxygen atom of the tetrahedral sheet. Depending on the type of association, phyllosilicates can be classified as 1:1 (also known as T:O) or 2:1 (or T:O:T) (Figure 1C).

**Figure 2:**
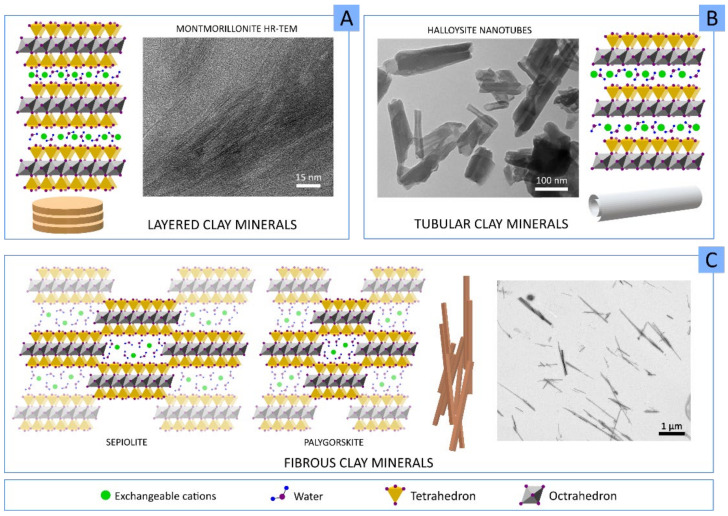
Structure and microphotographs of (**A**) layered clay minerals, (**B**) tubular clay minerals and (**C**) fibrous clay minerals. The SEM microphotograph of halloysite nanotubes (**B**) was adapted with permission from [12], Elsevier, 2021.

Although the most accepted classification of clay minerals usually depends on their structure and chemical composition (mineralogical classification) [11], in this manuscript, we mainly differentiate clay minerals by their crystal habits or macroscopic aspect (Figure 2). Therefore, we can differentiate between:

- Layered clay minerals such as montmorillonite (MMT), hectorite (HT), or laponite (LAP);
- Tubular clay minerals, where halloysite nanotubes (HNTs) are included;
- Fibrous clay minerals or “non-planar phyllosilicates”, a group formed by sepiolite (SEP) and palygorskite (PAL, also known as attapulgite).

In the particular case of layered clay minerals (Figure 2A), it is possible to find an “interlayer space” between each T:O or T:O:T stack. This interlayer space is of great importance since it provides clay minerals with useful properties. For instance, due to the interlayer space, they are able to “swell” in an aqueous environment, thus behaving as hydrogels at the proper concentration. This swelling behavior consists of the adsorption of water, leading to increased interlayer space and improvement in the volume occupied by the clay–water suspension. In fact, under optimum conditions, the layers can completely dissociate, leading to clay mineral delamination or exfoliation [13]. Inside this, we can find a quite famous synthetic clay called Laponite^®^. LAP is a synthetic clay mineral with a hectorite-like chemical composition whose name was introduced by Laporte Industries in the 1960s (currently named BYK Additives Ltd.) [14]. The use of LAP has historically focused on the chemical and construction industry mainly as a rheological additive for paints, household cleaning products, adhesives, automotive and wood coatings, ceramic glaze, etc. Nonetheless, its useful properties and its synthetic origin (which implies the absence of other mineral impurities typically accompanying natural clay minerals) have allowed its use to be extended to a wide variety of areas, including medicine or pharmacy [15]. Therefore, in comparison with its natural counterpart HT, LAP possesses a superior purity and very small particle size (20–50 nm diameter and 1–2 nm thickness), which allow it to produce transparent colloidal and gel-like suspensions maintaining the same rheological behavior as HT [16].

Halloysite is a very special clay mineral due to its particular idiosyncrasies. Halloysite is an isomorph of kaolinite, a layered clay mineral and member of the kaolin group, which can naturally adopt different morphologies. In fact, in the classification of Guggenheim and Martin, halloysite can be found in different groups depending on its final morphology: planar, rolled or spheroids [11]. In particular, halloysite nanotubes (HNTs) result from the curvature of newly forming halloysite silicate layers in the presence of water, thus forming the characteristic tubular morphology of this clay Figure 2B.

Fibrous clay has a continuous tetrahedral sheet, unlike the octahedral ones, which are discontinuous (Figure 2C). That is, SEP and PAL have continuous layers of tetrahedra, though these tetrahedra suffer inversions every six or four units (for SEP and PAL, respectively). As a result, tetrahedral apices point in opposite directions, forcing for a spatial modification of the octahedral sheet, which is discontinuous. These discontinuities create zeolitic channels within the structure. Coordination and charge balance are compensated along the channels by protons, water (zeolitic water and crystallization water) and exchangeable cations. The final crystal habit of SEP and PAL is fibrous or acicular, with zeolitic channels parallel to the fiber length.

In terms of rheology, layered clay minerals in aqueous environments possess “thixotropic” and “shear-thinning” behavior, which are the result of the combination of both swelling capacity and their pH buffering activity. These properties can also be obtained with fibrous clays, although in this particular case, the rheology will highly depend on the concentration and mixing technique [17,18].

**Figure 3:**
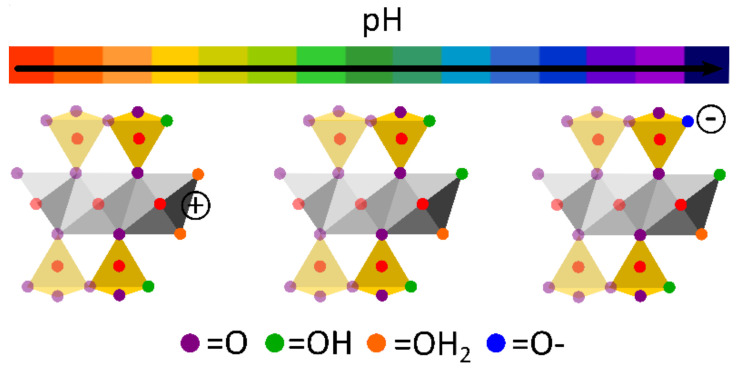
pH-dependent ion reactions at the edge of clay mineral layers.

Due to their chemical composition and crystalline structure, clay minerals have the ability to act as pH buffers. This property lies in the variations in the sign and density of charges located in the silanols groups at the edges (Figure 3). In an acidic medium, the excess amount of protons creates positive charges at the clay edge crystals, while negative charges are caused by the dissociation of silanols and aluminol groups due to alkaline environments. The knowledge of edge-charges of phyllosilicates is intimately related to the rheological properties. The widely known “house-of-cards” or “card-house” aggregation will occur when planar clay particles interact through the edge/faces of one another, thus producing shear-thinning or thixotropic rheological profiles [19].

Moreover, clay minerals possess negatively charged particle surfaces due to isomorphic substitutions. This charge imbalance is compensated by free cations in the media (Ca^2+^, Na^+^, K^+^, Mg^2+^, etc.), thus providing clay minerals with high adsorption capacity, highlighted by their high specific surface area. These properties were widely exploited for environmental remediation, catalysis and, more importantly for this review, drug delivery [13].

### 1.2. Clay Minerals in 3D Printing

**Figure 4:**
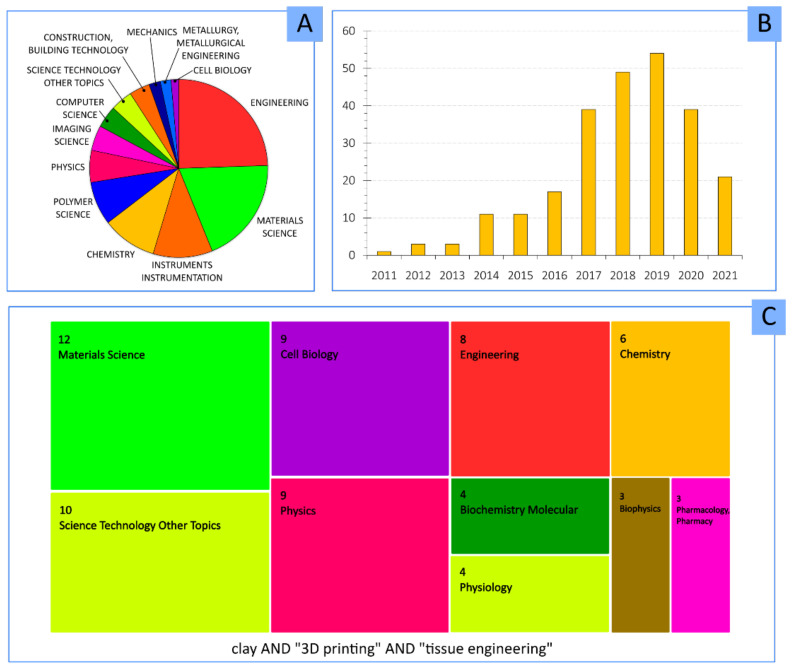
(**A**) Top 20 research areas with a higher amount of studies containing “clay” AND “3D printing”. (**B**) Number of publications per year containing “clay” AND “3D printing” keywords. (**C**) Main research areas for “clay” AND “3D printing” AND “tissue engineering”. Results obtained from database Web of Science. Current results in June 2021.

The combination of clay minerals and 3DP technology is a pretty recent field of study. According to the database “Web of Science”, the major part of publications belong to engineering, followed by materials sciences, chemistry and polymer science (Figure 4A). The addition of “tissue engineering” keyword (Figure 4C) centers the attention to materials science and cell biology. Apart from the novelty of 3DP, the combination of this technique with clay minerals makes it an exponentially growing field, as can be seen by the evolution of the number of publications per year (Figure 4B).

This section gathers those studies dealing with 3DP and clays. The studies discussed herein are somehow related to the biomedical field, or they could be of potential exploitation in the biomedical field, regardless of whether the clay is a part of the ink or acts as an “extra” or “support” ingredient for the 3DP process (Table 2). Layered clay minerals such as MMT and LAP are the most widely used in this field. Natural MMT was combined with high-density polyethylene (HDPE) in order to improve the polymer performance and create 3D printing filaments [20]. HDPE is a polymer with extended use in medicine, which was used as a bone substitute and orthopedic prostheses due to its remarkable biocompatibility and mechanical properties. Nevertheless, HDPE possesses inadequate rheological behavior, low modulus and poor bioactivity (bio-inert). Accordingly to the study by Beesetty and co-workers, the addition of 5% *w*/*w* of clay provided “superior mechanical performance”. Even if the final scope of this study was not related to medical applications, the fact that both ingredients (MMT and HDPE) are biocompatible allows these positive results to be potentially extrapolated to 3DBP and the production of different medical devices.

Organo-modified clays are clays that have been subjected to a specific type of surface functionalization. The organic functionalization of layered clay minerals is usually carried out by grafting the clay mineral with cationic surfactants or other organic molecules that are exchanged with the natural exchangeable cations present within the interlayer space of the clay. Therefore, the organic molecule enters the interlayer space, occupying the place of the exchangeable cations. Due to the larger size of these cationic surfactants, the interlayer space (d-space) of the clay expands, allowing other molecules to enter. Cloisite^®^ 30B (a natural MMT functionalized with alkyl-quaternary ammonium salt) was combined with polylactic acid (PLA), and the thermal and mechanical implications were studied [21,22]. PLA is a biocompatible and biodegradable polymer widely used in medicine [23,24]. Among its main properties, PLA possesses optical transparency and a high melting point (>150 °C), the latter property being incompatible with cell-laden 3DBP processes. Nonetheless, PLA is very useful for the production of tailor-made implants and medical devices, although the shape stability of the printed PLA objects still needs to be optimized. For instance, the shrinkage that occurs after PLA crystallization can alter the final shape of the 3D printed object, giving rise to warped pieces (low shape fidelity). In this regard, layered clay minerals such as MMT were proposed as PLA additives able to optimize printability and reinforce mechanical properties. In fact, Cloisite^®^ 30B enabled to increase the storage modulus and the crystallinity of PLA [22]. Moreover, the melting temperature needed for the printing process highly depends on the resultant architecture of the PLA/Cloisite^®^ 30B nanocomposite, which can be modulated by the clay mineral. The elastic modulus increased in all cases in which Cloisite^®^ was involved [21]. Other types of functionalized MMTs (Cloisite^®^ 5 and Cloisite^®^ 20) were also mixed with PLA, and their performance compared with a natural sodium MMT (Na Cloisite^®^). The aim was to explore the influence of these minerals during 3DP fused filament fabrication [25]. On this occasion, results clearly showed that the mechanical properties depend on the clay concentration and type. For instance, Paspali and co-workers reported that tensile moduli increased, while tensile strength and ductility reduced when MMT was used at 5% *w*/*w*. For the same clay concentration, the organo-modified samples (PLA/Cloisite^®^ 5 and PLA/Cloisite^®^ 20) reported better elasticity and strength than PLA/Na Cloisite^®^. This result was ascribed to the interlayer space of each MMT sample. The functionalized samples showed an increased interlayer space (d-space) due to the presence of the organic molecules, so these clay particles were more prone to intercalate PLA during the 3D printing process, thus explaining the better mechanical performance. Nonetheless, the Na Cloisite^®^ possessed the smallest d-spacing, hindering PLA intercalation [25].

**Table 2:** Clay minerals in 3DP technology with potential applications in medicine and TE.

Clay Mineral and Concentration	Other Ink Ingredients	Clay Role	3DP Technique	Ref
MMT (Cloisite^®^ 30B)—4% *w*/*w*	PLA	Increasing PLA crystallinity, melting temperature modifier and mechanical reinforcement	Fused deposition modelling	[21,22]
MMT (Cloisite^®^ 5, Cloisite^®^ 20, Na Cloisite^®^)—1, 5% *w*/*w*	PLA	Improved mechanical properties of PLA by organo-modified clay minerals due to increased d-spacing of organo-modified clay particles	Fused filament fabrication	[25]
MMT—0.5, 1, 2, 5% *w*/*w*	HDPE	MMT provided superior mechanical performance	Fused filament fabrication	[20]
MMT (Cloisite^®^ SE300)—1, 3, 5% *w*/*w*	PETG	Mechanical reinforcement. Simplification of the 3D construct post-processing or post-treatment	Extrusion	[26]
SEP—1, 2, 3, 5, 7% *w*/*w*	PETG	Improvement of mechanical properties due to directional alignment of SEP particles within PETG filament	Fused deposition modelling	[27]
LAP—2.5% *w*/*v*	Silk fibroin	Formation of a print-bed (in combination with PEG) to support silk-fibroin BI 3D constructs	Submerged extrusion into LAP–PEG suspension	[28]
LAP—7% *w*/*w*	NIPAAM, PAAM	Rheological modifier for 3DP and mechanical reinforcement	Extrusion	[29]
LAP—12% *w*/*v*	Pluronic	Improvement of printability and mechanical properties of ink used as sacrificial material template (mold) for the production of microfluidic system	Extrusion	[30]

In the case of synthetic clays, LAP demonstrated to favor the manageability of silk fibroin (SK) for 3D printing. SK is a biocompatible, mechanically robust polymer with high potential as a medical ingredient. The crosslinking of this polymer is subjected to the use of other additives, jeopardizing the resolution and the final shape fidelity of the constructs. In this regard, a recent one-step SF gelation was performed within a suspension of LAP and PEG; the latter acted as a printing medium in where SF was left to crystallize [28]. In this process, PEG induced SF crystallization, while LAP would be working just as a support and a rheology modifier. At the end of the process, LAP–PEG suspension was easily washed with PBS solution, and the SF construct was easily extracted. In a similar manner, LAP (12% *w*/*v*) was combined with pluronic (30% *w*/*v*) to be used as a template to produce a 3D microfluidic system for an organ-on-a-chip device [30]. Briefly, the process consisted of printing a template over which poly-dimethyl-siloxane (PDMS) organosilicon was added and cured. Then, the pluronic/LAP ink would be eliminated to reveal a three-dimensional, complex microfluidic system. To perform this, authors previously screened different pluronic/LAP concentrations in order to select the most printable ink able to support the pressure of PDMS afterward. In particular, the authors observed that high amounts of LAP were needed to obtain enough printability and shape retention, with filament collapse occurring below 8% *w*/*v* LAP. Nonetheless, a 12% *w*/*v* LAP concentration revealed the good performance of the ink. Concentrations over this value compromised the quality of the 3D template, probably due to the destabilization of pluronic micelles and too numerous clay–clay interactions, which make the mixture too rigid and low printable.

**Figure 5:**
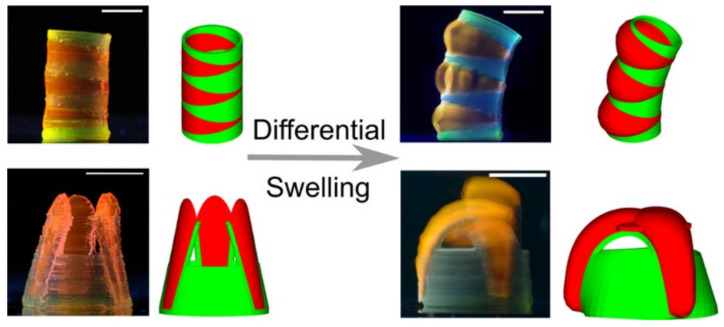
Shape-changing 3D constructs made of thermally responsive NIPAM/LAP swelling gel (red) and PAAM/LAP (green). Figure reproduced with permission from [29], American Chemical Society, 2019.

The production of medical implants and other medical devices with adjustable and tunable capabilities is of great interest. For instance, shape-changing constructs could be of great use for vascular implants, endoscopic applications, tissue engineering, etc. In this regard, N-isopropylacrylamide (NIPAM) and polyacrylamide (PAM) are synthetic gels with differential thermal swelling responsiveness [29]. These two ingredients were combined with LAP and proposed to produce 3D constructs with the ability to perform time-dependent shape changes (elongation, expansion, bending, gripping). These shape changes were based on the differential spatial combination of both NIPAM and PAM produced during 3DP (Figure 5). In both cases, LAP was used as a rheological modifier (to allow 3DP) and mechanical reinforcement.

**Figure 6:**
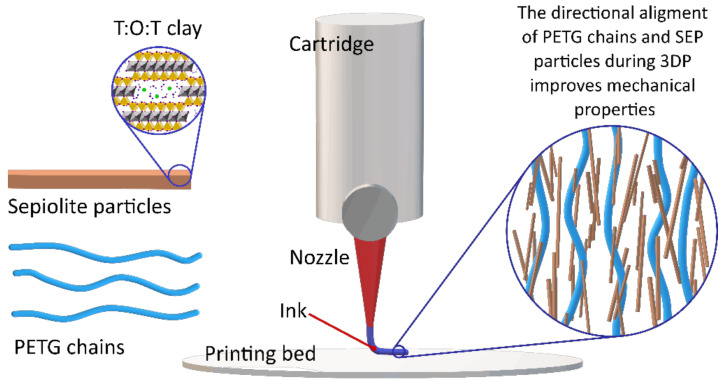
Schematic representation of the directional alignment of SEP particles with PETG chains during extrusion-based 3DP, according to the description and hypotheses reported in [27].

Finally, fibrous clay minerals (SEP and PAL) also demonstrated useful performances as additives for 3D printing techniques. Their high aspect ratio can be of great use in reinforcing 3DP polymers. For instance, SEP was incorporated into polyethylene glycol terephthalate (PETG) for fused deposition modeling 3DP [27]. The results are quite noteworthy and indicate that fibrous clay minerals could be of great use for the regeneration of tissues requiring high mechanical strength, such as tendons, ligaments, muscles or bones. Kim and co-workers added SEP to PETG in increasing amounts from 1 to 7% *w*/*w*. The tensile strength and the elastic modulus grew with increasing SEP concentration. These remarkable results were ascribed to the SEP particles directional alignment, parallel to the deposition direction during the 3D printing process (Figure 6). SEP particles’ alignment and their homogeneity within the formulation were addressed by means of electronic microscopy (SEM, TEM) and small-angle X-ray scattering (often abbreviated as SAXS). This way, SEP was confirmed as a great reinforcement of PETG polymer, something that could be applicable to other biocompatible polymers.

As it can be seen, clay minerals are remarkable ingredients to modulate the final mechanical properties of biocompatible materials with potential use in the fabrication of medical devices and implants. They can also optimize the printing process by being either added to the ink (where they can modify the rheology or the melting temperature of the rest of the ink ingredients) or by acting as supplementary/supporting materials during the printing process itself, such as for sacrificial material templates or printing beds.

## 2. Clay Minerals in 3D Bioprinting

**Figure 7:**
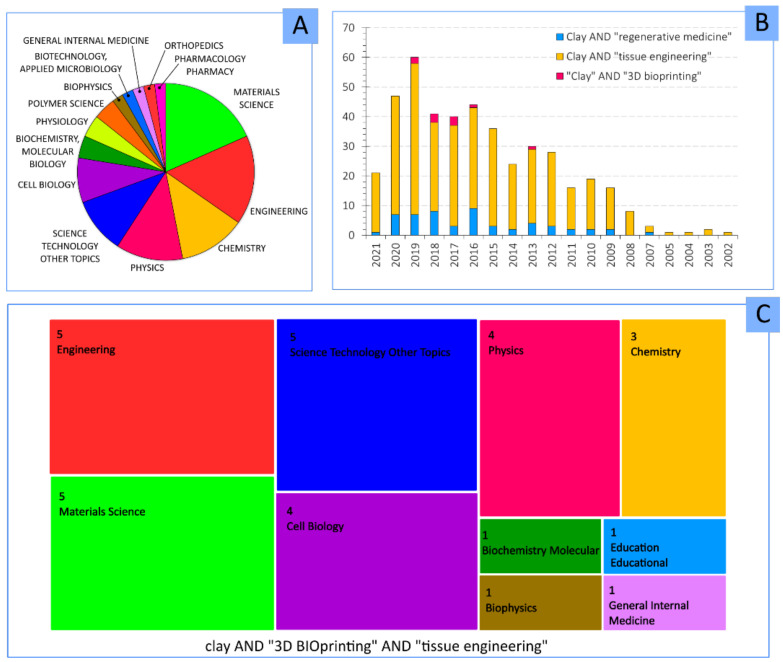
(**A**) Most important research areas of studies dealing with “clay” AND “3D Bioprinting”. (**B**) Number of publications per year containing “clay”, “3D bioprinting”, “regenerative medicine” and “tissue engineering” keywords. (**C**) Main research areas for “clay” AND “3D bioprinting” AND “tissue engineering”. Results obtained from database Web of Science, June 2021.

A lower number of publications can be found when “clay” and “3D bioprinting” keywords are combined. In fact, the scarce existing publications are centered in materials science, engineering and chemistry (Figure 7A,C), indicating that the addition of clays is more related to the mechanical and technical improvement for the 3DBP process. Despite that, the potential role and usefulness of clay minerals in 3DBP are supported by the increasing number of publications in which these inorganic ingredients were destined for regenerative medicine and tissue engineering scopes (Figure 7B).

The reasons for the usefulness of clay minerals in 3DBP are based on their properties and traditional uses. Throughout the years, clay minerals have proven themselves as useful rheology additives, showing shear-thinning behavior and viscoelastic profiles [16,31,32,33,34,35,36,37] and are even commercialized as such (i.e., different types of Laponite^®^ or Cloisite^®^ products series). In fact, this is a well-known feature: according to their structure, clay minerals such as SEP, PAL, MMT, HT and LAP are able to intercalate between polymer, polysaccharide and protein chains to modify their rheological and mechanical properties and provide crosslinking [3,27,38,39,40]. Moreover, clay minerals are naturally occurring materials whose biocompatibility and bioactivity have already been reported and reviewed [41,42,43,44,45,46,47,48]. The role of phyllosilicates as reinforcing materials for polymers and other ingredients was also demonstrated [26,49,50,51,52,53,54,55,56,57]. Additionally, polymer/clay nanocomposites were demonstrated to tailor cell detachment, cellular adhesion [58,59], cell-sheet formation [60] and to control the delivery of drugs and other bioactive molecules such as growth factors [61,62,63]. Moreover, natural clay minerals are naturally available resources with high availability and affordability. Therefore, it is reasonable to think that clay minerals will also be adequate and useful to improve BMI and BI formulations, awarding them with tunable rheological and mechanical properties as well as to contribute with certain bioactive properties. One of the objectives of this review is to offer an overlook of the most recent knowledge of clay minerals as ingredients of BMI (Table 3) and BI (Table 4) formulations and their roles during 3DBP and TE.

For a better understanding of the uses and/or potential uses of clay in 3DP, and ultimately 3DBP, it is necessary to clarify the final scopes of BIs and BMIs together with their most desirable characteristics enabling those scopes to be achieved. Precisely, the following section is dedicated to defining and classifying the most desirable properties of BIs and BMIs.

### 2.1. Desirable Bioink and Biomaterial Ink Properties

The success of 3DBP for TE lies in finding the right balance between a myriad of factors: biocompatibility, printability, shape fidelity, functionality and mechanical properties of the BI. Therefore, it is necessary not only to choose the BI or BMI ingredients carefully but also the optimal combination and concentration of each of them. Apart from this, the formulation performance also depends on the targeted tissue under regeneration, the cells included in it (if applicable), the 3DBP technique chosen and the required post-processing of the printed constructs. In other words, the key factor is the BI or BMI formulation.

#### 2.1.1. Printability and Shape Fidelity

**Figure 8:**
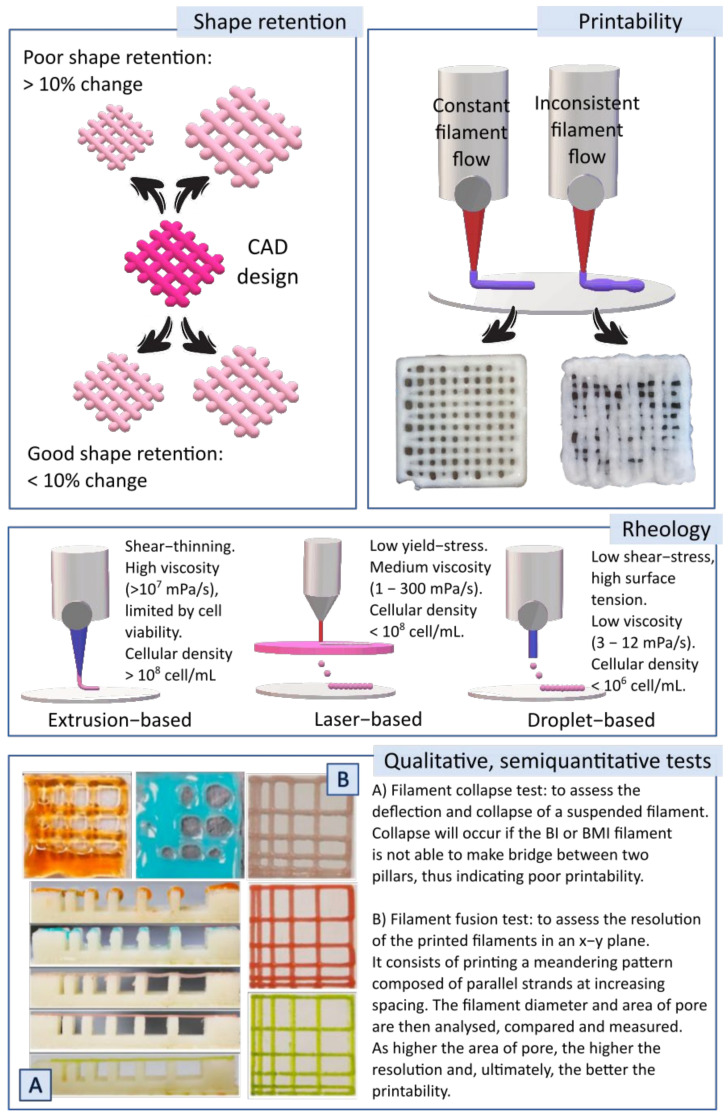
Up: schematic representation of “printability” and “shape fidelity” or “shape retention” concepts. Middle: summary of some desirable rheological properties of BI and BMI based on three 3DBP techniques (extrusion-based, laser-based and droplet-based). High viscosities are acceptable for extrusion-based techniques, although it is necessary to bear in mind that excessive viscosity could hinder cellular viability. Droplet bioprinting or laser-based techniques require low viscosity, though minimal values are needed to prevent cellular sedimentation. Down: qualitative and semi-quantitative tests used to evaluate printability and shape retention: (**A**) filament collapse test; (**B**) filament fusion test. Photographs for filament collapse and filament fusion tests were adapted with permission from [70], Elsevier, 2021.

In the determination of the appropriate properties of a BI “shape retention” (or “shape fidelity”) and “printability” are two concepts widely used. Shape retention or shape fidelity is, precisely, the ability of a BI to retain its shape after the bioprinting process. Those BIs whose 3D-printed constructs suffer from <10% change in length and width after reaching equilibrium (compared to the dimensions of the CAD design) are considered to have good shape retention. Printability can be defined as the ability of a BI or BMI to be continuously extruded at a constant printing speed and pressure [4,64]. These two properties highly depend on the rheology profile, which must be different depending on the 3DBP technique (Figure 8).

In general, for 3DBP, shear-thinning and viscoelasticity are crucial. A shear-thinning profile is shown by a material whose internal structure is temporally disrupted when subjected to stress forces such as shear and extensional flows (generated during processes such as 3DBP) but immediately recovered once the stress is removed. Thixotropic materials behave in a similar way, although the recovery of the material’s internal structure is time-dependent (it does not occur immediately after the stress removal) [3,4,65,66,67]. On the other hand, viscoelastic profiles are desirable to protect cells from shear stresses subjected during the printing process [68]. It is also worth mentioning the fact that cells are prone to alter the viscosity and rheology of the BI with respect to the corresponding BMI [69]. This modification of rheology produced by cells can be ascribed to the disruption of some interaction points between the BI ingredients (e.g., breakage of polymer–polymer interactions), something that would happen in a directly proportional manner to the cellular density of the BI. Consequently, this parameter must also be optimized. Apart from rheology, other qualitative tests can be used to measure printability and shape fidelity of BI and BMI. For instance, the filament collapse test (Figure 8A), filament fusion test (Figure 8B), pore geometry and lateral pore collapse are some examples [68,70].

#### 2.1.2. Biocompatibility and Functionality

**Figure 9:**
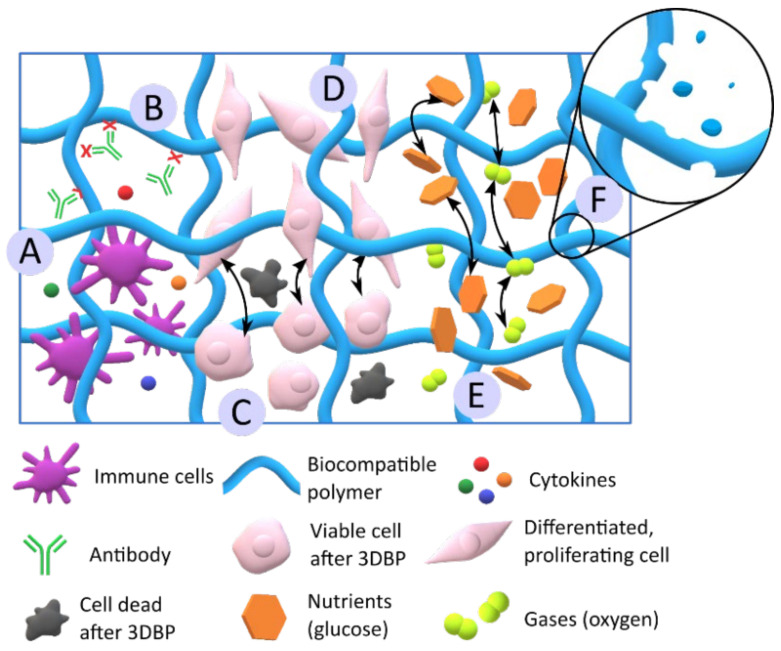
Schematic summary of the main desirable biological features of BIs and BMIs. (**A**) and (**B**) represent low immunogenicity of the BI or BMI, with low/absent cytokine release (**A**) and low antibody recognition (**B**). (**C**) Biocompatibility and cellular viability. (**D**) Cellular proliferation and differentiation. (**E**) The porosity of the BI and BMI allows for nutrients and gases diffusion throughout the 3D construct. (**F**) Construct biodegradability. It is worth highlighting that the degradation process must not result in the production of toxic metabolites.

Biocompatibility, non-immunogenicity and biodegradability are crucial for a BI or BMI to produce 3D constructs applicable in TE (Figure 9). Biocompatibility is essential to guarantee cellular viability (Figure 9C) [71,72,73,74,75,76]. The biocompatibility of 3D bioprinted constructs in vitro is usually determined by direct and indirect methods, following the ISO 10993-5 [77,78]. The absence of immunogenic response in vivo is important to ensure the construct’s safety. Therefore, the in vitro immunogenicity tests are very valuable in the early steps, which can be performed by determination of cytokine release/production [75,79,80].

The biodegradability of a 3D construct is desirable if total native tissue regeneration is obtained. It means that the three-dimensional cell-laden structure ultimately disappears from the treated area, being fully replaced by regenerated, totally functional native tissue. Moreover, the biodegradation rate is very important: the construct must be able to maintain its shape and function, at least until total recovery/regeneration. Consequently, the BI or BMI ingredients must be wisely chosen and combined according to their biodegradation rate [52,81,82]. Just as an example, hydrophobic and high crystalline polymers such as poly(ε-caprolactone) undergo very slow biodegradation [83]. The biodegradation of some ingredients, such as alginate, can also be induced by the addition of enzymes [84]. Likewise, gelatin degradation can also be controlled by the functionalization degree, such as the degree of methacrylation [85]. Regarding the monitoring techniques, the degradation rate of the BI or BMI is mostly determined by gravimetric techniques [76,77,86,87], although other methodologies such as rheology [88] or the detection of certain molecules or metabolites were also used.

The functionality of a BI or BMI can be understood as their ability to enable tissue regeneration, which implies the promotion of cellular proliferation, differentiation (Figure 9c,d), and new tissue formation in the long term [71,72,75,89]. Additionally, good porosity, both in the ink formulation and the resultant 3D construct, is also a guarantee of BI or BMI functionality. Sufficiently porous BI or BMI formulation and/or printed construct ensure the diffusion of gases (oxygen, carbon dioxide), nutrients and other active substances such as growth factors (Figure 9e). Usually, the porosity can be analyzed by microscopy techniques such as SEM [51,72,75] or by gravimetric/volumetric analysis [76]. The intimate relationship between porosity and mechanical properties can also be found. In fact, it was reported that higher construct porosity leads to smaller mechanical resistance [73], thus being crucial to find a compromise between them.

#### 2.1.3. Mechanical Properties

**Figure 10:**
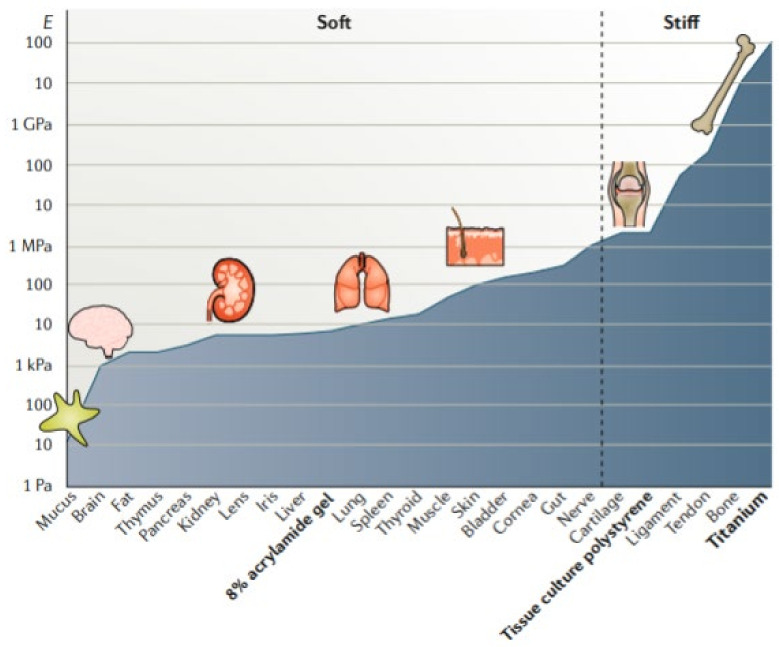
Crescent moduli of living tissues together with moduli of some TE materials. Tissues and materials are organized by increasing crescent moduli. Reproduced with permission from [106], Springer Nature, 2020.

The mechanical properties of BI and BMI must also be bore in mind, since they must address the particular characteristic of the targeted tissue. Especial attention must be paid if the BI or BMI is intended to substitute, repair or treat tissues involved in mechanical functions such as bones, tendons or muscles (Figure 10). Therefore, the resultant printed structures must support cells and newly formed tissue until total regeneration and resist different stresses and deformations without jeopardizing the construct integrity or the tissue functionality. Despite the numerous mechanical properties to which materials can be subjected (stress–strain, tensile, shear, bending and twisting), stress–strain and tensile tests are the most frequently addressed when it comes to TE 3D constructs.

Stress–strain tests are intended to subject the material to compressive forces. The compression modulus quantifies the ability of a material to withstand compressive loads. That is, it measures the ability of a material to change its length during compression. The lower the compression modulus, the higher the flexibility (the material is less stiff). By using a compressive test, it is possible to obtain different information about the elastic behavior of the material: Young’s modulus, the plastic yield point and the compressive strength [76,90,91,92,93,94,95].

**Table 3:** Biomaterial inks include clay minerals as part of their formula: clay type and concentration, final scope and main function(s) of the mineral within the BMI.

Clay Mineral	Clay Concentration	Other Biomaterial Ink Ingredients	Final Scope	Clay Role	Ref
**LAP**	10–100 µg/mL	GelMA	3D bone TE	Osteoinductive ingredient; controlled release of VEGF	[85]
	3, 4 and 5% *w*/*v*	GelMA	Printability studies of BMI for TE (target tissue undetermined)	Increase in porosity and printability	[96]
	Not specified	GelMA	3D skeletal muscle TE	Carrier and control release of VEGF	[63]
	5% *w*/*w*	PEGDA, ALG	3D cartilage TE	Improved printability and shape retention	[97]
	6% *w*/*v*	PEGDA, ALG, GEL	Development of self-supporting BMI for complex, in air, 3D structures	Printability and shape retention: self-supporting ingredient. Enabled “printing-then-crosslinking” process; Improved mechanical properties; Control of construct biodegradability	[40]
	0.1, 0.5 and 1% *w*/*v*	ALG-GEL	3D TE (target tissue undetermined)	Optimization of BI material printability	[98]
	4.5% *w*/*w*	AGA, PAM	3D TE (target tissue undetermined)	Mechanical reinforcement ingredient	[99]
	6, 12, 18 and 22% *w*/*w*	NIPAM, CNT	Medical device for human motion monitoring	Mechanical reinforcement ingredient; Biocompatible to fibroblasts	[7]
	10% *w*/*w*	PAAM, PEDOT	Medical device for neurological regeneration	Improved conductivity and mechanical properties	[100]
	7–9% *w*/*w*	HEMA	3D scaffolds able to direct cellular attachment, growth and differentiation	Improvement and modulation of cellular attachment and motility	[101]
	1.4–1.7% *w*/*w*	PMet-b-POxa	Stimuli-responsive BMI for TE (target tissue undetermined)	Modification of polymer gelling temperature, improvement of shape-fidelity	[102]
	6 and 10% *w*/*w*	SPE	Medical device for lower limb prostheses adapted to movement	SPE crosslinker, rheological additive	[103]
	0.1, 0.5 and 1%	TEMPO BC, ALG	3D TE (target tissue undetermined)	Printability and shape-fidelity enhancer; control release of BSA	[104]
**HT and LAP**	0.5–7% *w*/*v*	PEGDA	Recyclable 3D construct for biocatalysis	Rheological additives: HT induced higher viscosities with lower shear-thinning profile with respect to LAP. HT performed higher printing fidelity and faster construct biodegradation	[81]
**HNTs**	2, 3, 4, 5% *w*/*v*	ALG, MC, PVDF	3D cartilage TE	Improved mechanical properties	[91]
**PAL**	50–90% *w*/*w*	PVA	3D bone TE	Osteoinductive ingredient with significant mechanical resistance	[90]
**Cloisite^®^ Na**, **Cloisite^®^ 30B**, **Cloisite^®^ 15A**	3% *w*/*w*	GelMA	Production of 3D bioactive medical devices	Rheological additives; improved and controlled porosity. Mechanical reinforcement ingredients	[105]

The tensile properties of a material are quantified by tensile tests. In particular, tensile strength measures the ability of a material to withstand lengthwise stresses without fracture; thus, the maximum amount of tensile forces that a material can withstand before failure. In relation, the elongation at break is the length increase that the material will acquire during a tensile strength test before breaking [21,27,91].

### 2.2. Use of Clay Minerals as Printability and Shape Fidelity Ingredients

Natural and synthetic clay minerals proved their useful and tunable rheological properties through the years. These inorganic ingredients were combined with a wide variety of natural and synthetic polymers and macromolecules in an attempt to improve their rheology or their printability properties. Even though it seems simple, the optimization of printability by clays requires to consider several factors: the clay particle size, the possible interactions (covalent and non-covalent) with the rest of the ingredients, the clay concentration, the homogeneous dispersion of clay particles, the influence of the clay during the post-processing steps and time-dependent rheological changes due to clay swelling, among others. This section is devoted to the effects and influence of clay minerals as printability ingredients of different BI and BMI in order to understand their roles.

**Figure 11:**
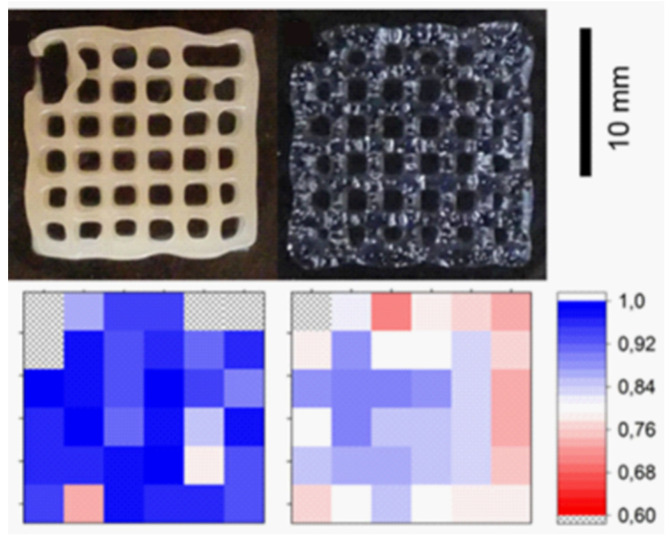
Shape fidelity of the printed PEGDA lattices using HT (left) and LAP (right) as rheological additives. The bottom row shows the shape fidelity of the comparison with the desired structure, graded with a color code. Blue shades depict a high accuracy or printing; Red indicates low conformity. Areas with printing errors are marked with “x”. Reproduced with permission from [81], Wiley Online Library, 2018.

Among clay minerals, there are not only differences in particle shape but also in particle size, something that greatly affects the rheological behavior of clay suspensions. The studies on the influence of clay particle size on BI and BMI printability are currently scarce. Nonetheless, Schmieg and co-workers highlighted this factor when they compared the rheology and printability of a natural, purified HT (commercialized as Bentone^®^ MA) with Laponite^®^ RD (LAP) [81]. The final scope was to optimize the 3DBP of an enzyme-laden poly(ethylene glycol) di-acrylate (PEGDA) BMI, meaning that no cells were added to the formulation. HT induced higher viscosity than LAP but lower shear-thinning behavior. This means that, when dealing with BI (cell-laden BMI), LAP would allow for better cellular viability results. The higher shear-thinning behavior of LAP-based systems can be ascribed to the smaller size of LAP with respect to HT. Nonetheless, the quality control of the printed lattice structures (Figure 11) revealed that the HT/PEGDA hydrogel retained the highest printing fidelity.

**Figure 12:**
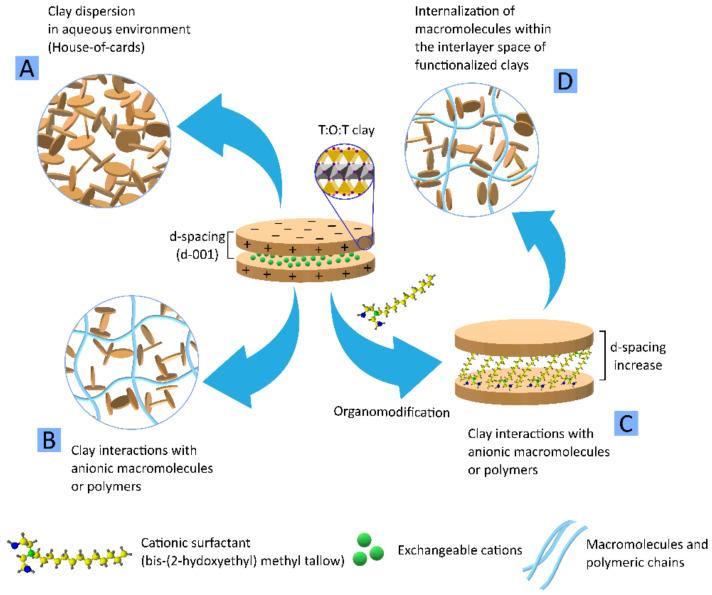
Schematic representation of layered clay nanoplatelets structure and charges (central). (**A**) House-of-cards interactions of clay nanoparticles in aqueous suspension after proper, homogeneous dispersion. (**B**) Clay particles interactions with anionic macromolecules in an aqueous environment. The clay–polymer–clay interactions mediate the crosslinking process. (**C**) Functionalization/organo-modification of layered clay mineral with an example of cationic surfactant. (**D**) Schematic representation of neutral/cationic macromolecules “sandwiched” between layered clay nanoplatelets. The increase in d-spacing after organo-modification makes clay particles more prone to intercalate molecules such as polymers and macromolecules.

As previously mentioned in the introduction, clay particles possess a negative net charge in the interlayer space due to isomorphic substitutions. This, together with the silanol groups placed in the edges of the particle, makes them useful not only as rheology modifiers but also as ionic crosslinking agents (Figure 12). For instance, carboxymethylcellulose (CMC) and sodium alginate (ALG) was combined with MMT [70]. The resultant printability of the BI was ascribed by authors to the establishment of intermolecular hydrogen bonds and electrostatic attractions between CMC, ALG and MMT. Figure 12b represents the possible electrostatic interactions between clay nanoparticles and other negatively charged polymers such as CMC and ALG.

A PEGDA/ALG BMI is able to produce stretchable and tough 3D constructs intended for cartilage tissue regeneration [97]. Nonetheless, the viscosity of the pre-gel solution (that is, the BMI before crosslinking process) needed to be improved in order to obtain enough shape fidelity. In that sense, the addition of 5% *w*/*v* of LAP provided enough viscosity and a shear-thinning profile. The self-supporting ability provided by LAP to PEGDA was also explored with ALG and GEL [40]. In fact, PEGDA/LAP, ALG/LAP and GEL/LAP 3D constructs were successfully printed in air, retaining their shape throughout the whole printing process. In other words, it was possible to print the whole construct and perform the corresponding chemical or physical crosslinking (which will award the manageability) afterward: “printing-then-crosslinking”. The self-supporting capability of 6% *w*/*v* LAP was proved by the ability to print high, complex structures with inclination angles of 45°. Although Jin and co-authors reported that LAP nanoparticles participate in the PEGDA, ALG and GEL crosslinking, the presence of any of these ingredients hindered the thixotropy and yield stress values provided by LAP suspension [40]. This was probably related to the concentration of the ingredients and the preparation procedure of the BMI. Indeed, it was reported that excessive ALG concentrations could hinder the viscosity provided by LAP due to excessive interactions between the anionic molecules of ALG and the positive charges of LAP nanoparticles, preventing the formation of the house-of-cards clay hydrogel network [107]. Another example of highly complex 3D constructs produced thanks to the participation of LAP was reported for a BMI formulated with 0.5% LAP and 2,2,6,6-tetramethylpiperidinyl-1-oxyl-oxidized bacterial cellulose (TEMPO BC) with ALG [104]. Precisely, they were able to obtain nose and ear flexible constructs thanks to the remarkable printability and mechanical properties of the resultant BMI.

**Figure 13:**
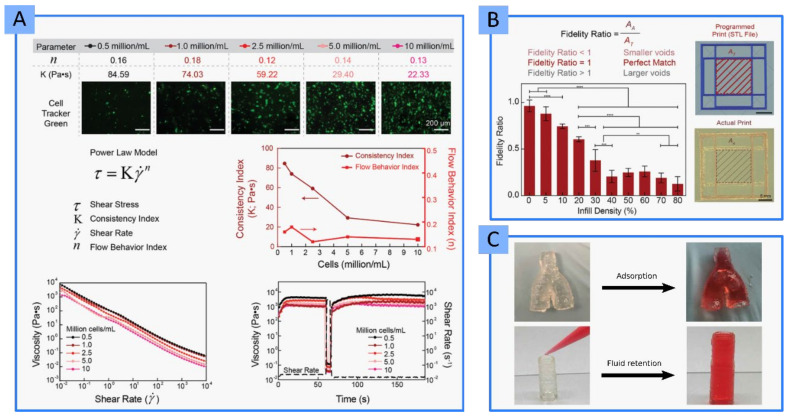
(**A**) Rheological parameters of GelMA/PEGDA/LAP BI loaded with different cellular densities. (**B**) Dependence of fidelity ratio with respect to the infill density (%) of the printed constructs. ** *p* < 0.05, *** *p* < 0.005, and **** *p* < 0.0001. (**C**) Some interesting behaviors of the printed structures after 3DBP and curing. The formulated BI allows obtaining complex 3D free-standing constructs that are able to absorb and retain fluids, something of great utility for vascular designs. Reproduced with permission from [69], Wiley Online Library, 2021.

Very recently, different BIs made of PEGDA, gelatin methacryloyl (GelMA) and LAP (and their combinations) reported improved printability and high fidelity after the addition of the nanoclay [69]. In fact, after LAP addition, the BIs were able to bridge a gap, something that GelMA and PEGDA were not able to achieve. As it is well known, LAP provided shear-thinning profiles and improved yield stresses. High yield stresses provide more control over the deposition of the BI and, therefore, higher printability. Furthermore, this particular study demonstrated that (i) the consistency of the final BI decreases after the addition of cells (Figure 13A), which disrupt polymer–polymer and polymer–LAP interaction points, and (ii) the printability and shape fidelity is highly dependent on the infill density (Figure 13B). In fact, this BI was used to obtain a multicellular, free-standing three-dimensional vascular model (Figure 13C) able to provide a “potential tool to understand vascular disease pathophysiology” among others [69].

Different LAP concentrations (3, 4 and 5% *w*/*v*) were combined with GelMA to create a BMI with acceptable printability [96,98,108]. As expected, the printability increased with LAP concentration, as well as viscosity. In this case, the printing fidelity should be sacrificed in cell-laden GelMA-LAP hydrogels since high viscosity would favor printability, but it will also put at stake the cellular viability [96]. LAP also improved the printability of oxidized alginate–gelatin (ALG-GEL) hydrogel [98]. More precisely, LAP increased the viscosity of the BMI over time, probably due to the adsorption of anionic groups of gelatin with the positively charged edges of LAP, leading to a crosslinking process. Authors also speculated that NH_3_^+^ groups of gelatin could be interacting with the negatively charged surfaces of exfoliated LAP particles, thus contributing to the hydrogel crosslinking. These authors also reported the modification of the hydrogel pH due to LAP. The ability of clay minerals to act as pH buffers lies in the variations in the sign and density of charges located in the silanols groups at the edges (Figure 3). When working with BMI and BI, this property of clay minerals should be taken into account since it could trigger either desirable or undesirable chemical reactions (polymer crosslinking) or affect cellular viability. In particular, Nerger and co-workers published the difficulties found during the 3D micro-extrusion of type I collagen when combined with LAP [64]. Collagen is a fibrous component of the ECM, its fibers serving as a scaffold and mechanical support of the tissue. In vivo, collagen fibers are self-assembled, something that is not easy to accomplish in vitro. The aim was to obtain anisotropic type I collagen 3D cell-laden constructs. Thus, the addition of different shear-thinning ingredients was explored, among which LAP was selected. The problem was that LAP and collagen were not compatible, resulting in a heterogeneous BMI that frequently clogged the printing nozzle due to collagen aggregates. Authors ascribed this incompatibility to the charged particles of LAP, which would be hindering the self-assembly process of collagen fibers [64]. Notwithstanding this hypothesis, the buffer activity of LAP particles must also be considered. In aqueous media, the PZC (point of zero charge or isoelectric point) of LAP particles is around pH 11 [109]. This means that LAP, according to its buffer activity, would tend to raise the pH values or dissolve within the acidic environment. If we take into consideration that collagen dissolves at acidic pH and crosslinks in an alkaline environment, this could be another possible explanation for the incompatibility reported by Nerger and co-workers: LAP could be causing a change in pH that would trigger abrupt collagen crosslinking, preventing fibers from alignment and jeopardizing printability.

Increases in aqueous clay concentration usually lead to an increase in viscosity, which is, in theory, desirable for extrusion-based 3DBP. Nevertheless, the nature and complexity of BMI and BI make it difficult to predict how the clay concentration will affect the final printability. Several studies revealed the necessity of systematic studies on this matter. For instance, the rheology studies performed by Peak and co-workers revealed that “increasing LAP concentrations decreased the recovery time due to incomplete destruction of internal house-of-cards structure” [74]. Similarly, the higher the LAP concentration, the higher the storage modulus of the hydrogel. Therefore, a rapid viscosity recovery was found for LAP–PEG in comparison with pure PEG BI, which is undeniably desirable for bioprinting. The high printability of this BI was demonstrated by a 23 layers-tall construct, printed without any other support material. Very interesting was the conclusion that LAP < 4% *w*/*v* (for a 10% *w*/*v* of PEG) does not allow sufficient structure recovery, so the concentration should be increased [74]. In a similar study, LAP and PEGDA were combined to serve as support for a hyaluronic acid (HA) cell-laden BI [71]. The necessity of LAP concentrations higher than 4% *w*/*v*, when combined with PEG, was further confirmed by Zhai and co-workers, which literally stated: “when less than 7% *w*/*v* nanoclay was added to the 20% PEGDA solution, the printed scaffold could not maintain a fixed shape with fused microstructure” [71]. The highest concentration limit was established to be 7% *w*/*v*, since higher LAP amounts are reported to require excessive pressures at room temperature. PEGDA was recently combined with de-cellularized cardiac extracellular cardiac matrix (dcECM) to obtain a BI intended for cardiac 3DBP TE [92]. The use of dcECM is a guarantee for biocompatibility and cellular viability of the resultant constructs, but the printing process, the myofibroblasts differentiation and the construct performance after implantation are compromised due to the poor mechanical performance of dcECM. Therefore, LAP was one of the selected ingredients (together with PEGDA) that helped to improve the rheology, printability and structural stability of the BI [92]. Thus, poly(ethylene glycol)-dithiothreitol (PEGDTT) is a PEG able to be hydrolytically degraded and, therefore, more convenient for TE purposes. Nonetheless, it shows too low viscosity on its own, limiting its use in 3DBP. The addition of 4% *w*/*v* of LAP to PEGDTT made it possible to increase the viscosity and provide a shear-thinning profile, thus improving printability and shape-retention [82].

**Figure 14:**
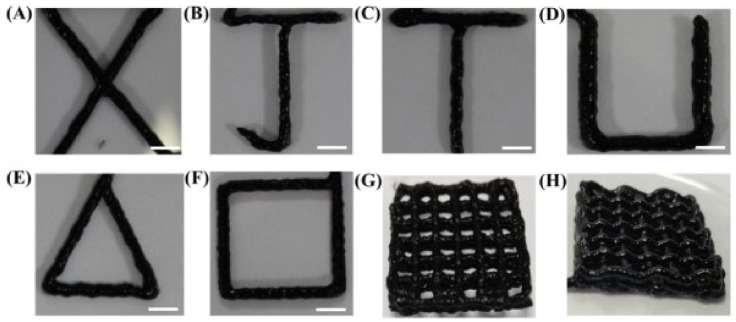
Three-dimensional printing behavior of different NIPAM/LAP/CNT BMIs (**A**–**H**) reported by Deng and co-authors. Adapted with permission from [7], American Chemical Society, 2019.

N-isopropyl acrylamide (NIPAM), carbon nanotubes (CNT) and LAP were used to formulate conductive, stretchable and adhesive hydrogels. The authors of this document claimed that the printability of the NIPAM/LAP/CNT was appropriate [7]. Nonetheless, the filament definition was arguable due to lumpiness (Figure 14). Moreover, some filament collapse was also visible. It is worth mentioning that the authors did not specify the amount of LAP used, thus not being possible to discern how the clay mineral was influencing the printability.

The structure of clay minerals and, especially their specific surface area, makes them able to absorb high amounts of water. This happens at different speeds depending on clay type and concentration and influences the rheology of the resultant suspension. Naturally, this factor will influence the printability of BMIs and BIs. Consequently, it is of crucial importance to discern if the formulation rheology modifies with time in order to perform the printing process in due course. This phenomenon was recently observed by Sällström and co-workers, who added LAP to the photo cross-linkable N-(3-Sulfopropyl)-N-methacroyloxyethyl-N,N-dimethylammonium betaine (SPE) [103]. The SPE/LAP suspensions displayed low viscosities and Newtonian behavior just right after their preparation (pre-gel), whereas higher viscosities and shear-thinning profiles were found 24 h later. Therefore, the so-called pre-gel was considered not suitable for extrusion-based printing, requiring 24 h of aging [103]. This result brings to the fore the importance of clay suspensions “aging” or “maturation”. It is typical for clay minerals, especially for layered ones, to undergo physicochemical changes with time after being dispersed in water [17,18,107,110,111]. Among them, rheological changes are of special importance for 3DP and 3DBP. The major part of short-time rheological modifications of clay suspensions can be ascribed to their swelling capacity and high specific surface area. With time, the dispersion of clay nanoparticles improves, exposing a higher surface area and, therefore, increasing the viscosity. As an example, Jin and co-workers prepared LAP suspensions 24 h prior to being used as BMI ingredient, thus allowing the clay hydrogel to set and swell properly [40]. This procedure could be interesting to reduce the required amount of clay in BI or BMI by simply waiting for it to age until maximum consistency.

The 3DBP process includes not only the printing itself but also the post-processing of the construct to make it a structurally stable and manageable piece. Clay minerals have also proven to be able to sway the post-processing of the printed construct. In this sense, some polymers undergo gelation by physical mechanisms such as temperature changes. In fact, during 3DBP, the temperature is a key factor since it will influence the rheology and, more importantly, cellular viability. This factor is even more important when working with thermoresponsive ingredients such as GEL and GelMA, among others. In these cases, printability highly depends on the cartridge’s operating temperature, the print-bed and the environment, as well as on the speed with which the thermoresponsive ingredient changes from a solid-like to liquid-like state. The gelation temperature of thermoresponsive ingredients can vary with the addition of clay minerals, as it was reported for kappa-carrageenan (k-CA), agarose (AGA) and the block copolymer poly(2-methyl-2-oxazoline)-b-poly(2-n-propyl-2-oxazine) (PMet-b-POxa) [102,112,113].

**Figure 15:**
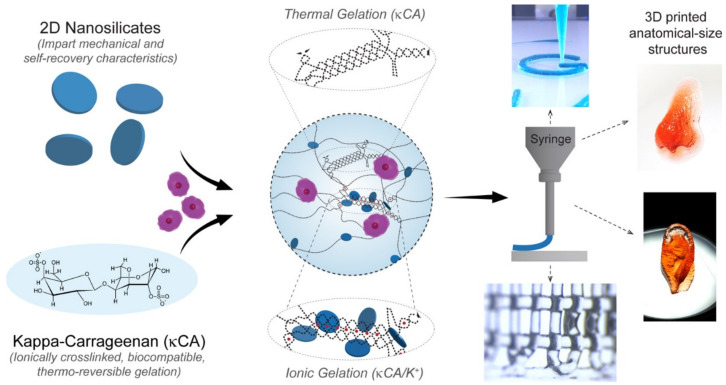
Interactions between LAP and k-CA polymer in a BI. Schematic representation of the crosslinking process of the thermoresponsiveness and ionic gelation. Adapted with permission from [113], American Chemical Society, 2017.

Regarding k-CA, its gelation temperature (around 40 °C) compromises the printability of cell-laden inks. The addition of LAP to k-CA improved the printability of this polymer, allowing the preparation of a suitable BI [113]. In particular, in the presence of 2–2.5% *w*/*w* of LAP (LAP/k-CA composite), the k-CA gelation temperature was thoroughly reduced to 35 °C (Figure 15). Moreover, the yield stress and pressure required for the k-CA/LAP BI extrusion was reduced by the clay mineral, while the recovery time of the final k-CA/LAP BI was increased by the synthetic nanoclay. This was due to disruptions of the polymer–polymer entanglements by interference with LAP nanoparticles. In conclusion, at least 4% of LAP must be combined with 2.5% of k-CA in order to obtain a printable k-CA BI [113].

Another example of a thermoresponsive polymer is AGA, which highlights by its thermal-reversible sol–gel transition, its biodegradability and biocompatibility. Nonetheless, it is a very complicated polymer to obtain good shape-fidelity constructs in 3DBP. This difficulty is mainly related to the time required by the polymer to set once printed: AGA setting requires rapid temperature changes. It was reported that the combination of AGA, PAAM and LAP could be successfully used to obtain complex structures through extrusion 3DBP [99]. Further changes in gelation temperature of AGA by LAP can be found. Topuz and co-workers combined AGA and LAP to prepare fibroblast-laden nanocomposites [112]. On this occasion, no 3D printing was performed, although authors reported that the gelling temperature of AGA increased after LAP addition due to the chemical interactions between the polymer and LAP surface. In particular, the gelation temperature of the AGA used in this study shifted from 25 °C for pure AGA to approximately 37 °C when mixed with 4.5% *w*/*v* of LAP [112]. Therefore, it is crucial to optimize the effect of clay minerals over thermoresponsive ingredients since it helps to select adequate conditions for 3DBP.

PMet-b-POxa behaves as a gel at temperatures higher than 20 °C, while sol behavior can be found for temperatures below this value. The addition of LAP (1.4–1.7% *w/w*) was reported to slightly alter the gelation temperature of the polymer [102], in agreement with previous AGA observations reported by Topuz [112]. Printed strand fusion was suppressed due to the presence of LAP, meaning that the shape fidelity was effectively improved. Moreover, smoother and more uniform BMI strands were also obtained after the addition of the synthetic clay mineral. The authors explained this result due to the strengthening of the overall structure of the hybrid hydrogel as evidenced by an increase in the elastic modulus (G′). Nonetheless, it is worth mentioning that high pressures were used during the printing process (110–220 kPa), which could limit the use of this formulation as BI due to potential cellular viability jeopardizing [102].

Functionalized or organo-modified natural clay minerals were used in BI and BMI formulations. An organo-modified clay is a clay that has been subjected to a specific type of surface functionalization. The organic functionalization of layered clay minerals is usually carried out by grafting the clay mineral with cationic surfactants or other organic molecules that substitute the natural exchangeable cations present within the interlayer space of the clay. Under optimal conditions, the organic molecule enters the interlayer space of clay nanoplatelets, occupying the place of the exchangeable cations. Due to the larger size of these cationic surfactants, the interlayer space of the clay expands, allowing for other molecules (e.g., polymers) to enter. An example of organo-modified or functionalized clay minerals is Cloisite^®®^ 30B, which were combined with bis-(2-hydroxyethyl) methyl ammonium tallow molecules (Figure 12C).

**Table 4:** Bioinks, including clay minerals in their formula: clay type and concentration, final scope, and cellular viability.

Clay	Clay Concentration	Rest of Bi Ingredients	Final Scope	Cell Type	Cellular Viability	Ref
**LAP**	3% *w*/*w*	ALG, MC	3D skeletal tissues engineering	Mesenchymal stem cells	70–75%	[73]
	3% *w*/*v*	ALG, MC	3D bone TE	Human bone marrow stromal cells	>90% from day 7	[114]
	0.5% and 1% *w*/*v*	GG	3D bone TE	Myoblasts	80%	[115]
	2.3% *w*/*w*	dcECM, PEGDA	3D cardiac TE	Human cardiac fibroblasts	>97% in 7 days	[92]
	0.5, 0.75, 1 and 2% *w*/*w*	GelMA	3D bone and vascular TE	Human bone marrow stromal cell	85% in 21 days	[72]
	1–5% *w*/*w*	GelMA/PEGDA	Production of multicellular, free-standing 3D vascular model	Endothelial cells and vascular smooth muscle cells	>85% post-extrusion on days 1, 3, and 7	[69]
	7% *w*/*v*	NAGA	3D bone TE	Osteoblasts	Not mentioned	[89]
	>4% *w*/*v*	PEG	3D TE (target tissue undetermined)	Pre-osteoblasts	>90% in 21 days	[74]
	7% *w*/*v*	PEGDA, HA	3D bone TE	Osteoblast	95% after 1 day	[71]
	0–6% *w*/*v*	k-CA	3D TE (target tissue undetermined)	Pre-osteoblasts	Not reported	[113]
	4% *w*/*v*	PEG and PEGDTT	Control and direction of cell migration	HUVEC	85% just after 3D bioprinting	[82]
	0.1, 0.25, 0.5 and 1% *w*/*v*	AGA	Increase the bioactivity of AGA BI	NIH/3T3 fibroblasts	Analysis performed, quantitative data not specified	[112]
**MMT**	4% *w*/*v*	CMC, ALG	3D soft-tissue engineering	Human pancreatic cancer cells	84% after 7 days	[70]

Cloisite^®®^ 30B together with Cloisite^®®^ 15A and sodium Closite^®^ were studied as ingredients of GelMA. GelMA is a photo-cross-linkable macromere with adequate biocompatibility to be used as an implant. In fact, GelMA properties were compared to gelatin, thus being its potential substitute in 3DBP. Nevertheless, the low viscosity of GelMA and the time required to be adequately cross-linked, difficult the printing processes. The addition of the three types of commercial MMT (Cloisite^®®^) was reported to provide shear-thinning behavior and increasing viscosity of GelMA, enabling the use of the resultant BMI for TE purposes [105]. The highest viscosity was provided by a non-functionalized sodium Cloisite^®®^, in comparison with the two functionalized counterparts (Cloisite^®^ 30B and Cloisite^®^ 15A). This manuscript is of special interest since it highlights another important factor influencing the printability of BI and BMI with clay minerals in their formulation. When it comes to clay minerals, a proper dispersion of the individual clay nanoparticles could make a huge difference between a printed construct with acceptable or non-acceptable 3DBP performance. In this regard, the printability of GelMA hydrogels reported by Alexa and co-authors [105] was improved by the three of the MMT used with respect to pure GelMA. Nonetheless, the functionalization of the clay particles (Cloisite^®^ 30B and Cloisite^®^ 15A) proved to hinder the homogeneous dispersion of the clay particles within the GelMA matrix, thus clogging the printing nozzle occasionally and creating discontinuous filaments.

### 2.3. Clay Minerals as Biocompatible and Functional Ingredients of Bioinks and Biomaterial Inks

#### 2.3.1. Biocompatibility, Cellular Adhesion and Proliferation

Although the presence of an excessive amount of clay particles may be a problem for cellular viability due to precipitation and endocytosis [41,116,117,118,119], various studies have shown that certain amounts of clays in BI and BMI could positively impact the biological performance of the constructs by many reasons.

Hong and co-workers added LAP to a PEGDA/ALG BI with the main objective to improve printability [97]. Nevertheless, they discovered that the presence of the inorganic ingredient gave higher viability of human embryonic kidney (HEK) cells (95% viability) compared to the corresponding formulation without LAP (75% viability). A k-CA/LAP BI loaded with pre-osteoblasts proved good biocompatibility, as indicated by the even distribution of cells within the 3D construct. Likewise, the cellular viability remained constant for 7 days, something ascribed to the rheological properties provided by LAP [113]. Thus, LAP has proved a good rheological ingredient to protect cells from suffering during the 3D bioprinting process of a BI made of PEGDA/GelMA/LAP, with rather high cellular density (0.5 million/mL–10 million/mL) [69]. This conclusion was obtained after determining that cellular viability was >85% during the post-extrusion days (Table 4). Jin and co-authors worked with one of the highest LAP concentrations (6% *w*/*v*) added to PEGDA, ALG and GEL [40]. Despite that, the constructs reported good biocompatibility, cellular adhesion and proliferation. In fact, the cellular adhesion was improved by LAP, which provided “focal points” for the cells to adhere to.

**Figure 16:**
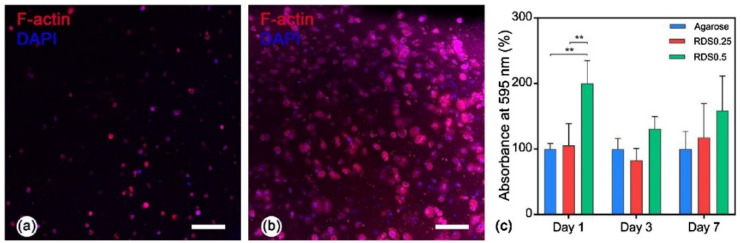
(**a**) Growth and cytoskeleton of fibroblasts cultured for a week in AGA gel; (**b**) growth and cytoskeleton of fibroblasts cultured for a week in AGA/LAP BI containing 0.25% (*w*/*v*) of clay mineral; (**c**) Metabolic activity of fibroblasts cultured within three different BI: pure AGA, AGA/LAP with 0.25% and 0.5% (*w*/*v*) of LAP (mean ± SD, ** *p* < 0.01, *n* = 3). Reproduced with permission from [112], Elsevier, 2018.

Bio-inert polymers are those which do not initiate a response or interact when introduced to biological tissue. This is of great use when it comes to avoiding immune responses, but it is detrimental for cellular adhesion and proliferation since it hinders the stimulation of tissue regeneration. In some studies, clay minerals were reported to improve the biocompatibility of some ingredients such as AGA, which forms a bio-inert hydrogel. With this specific scope, Topuz and co-workers formulated a BI made of LAP and AGA [112]. NIH/3T3 fibroblasts were encapsulated in the AGA/LAP hydrogels, the confocal microscopy analysis revealing cellular morphology maintenance, enhanced cellular proliferation (higher metabolic activity) and spreading (Figure 16), something that did not happen for pure AGA constructs. By incorporating LAP to 2-hydroxyethyl methacrylate (HEMA), positive charges were introduced to a protein-free material for 3DP [101]. LAP was able to confer direct fibroblasts attachment along the 3D construct.

Human pancreatic cancer cells (BxPC3) printed within a CMC/ALG/MMT BI performed an 84% cellular viability after 7 days [70]. This viability was reported to be higher than some other BIs formulated with similar ingredients, indicating that the addition of 4% of sodium MMT has a remarkable potential to enhance biocompatibility and cellular viability.

The in vitro viability and cell density of C2C12 pro-myoblasts increased for 21 days in a very similar manner for the 3DBP construct made of gellan gum (GG) with or without LAP [115]. Nevertheless, the implantation of the GG/LAP in the chorioallantoic membrane of a 10-day-old developing chick produced a remarkable vasculature infiltration with respect to the corresponding control.

A NIPAM/LAP/CNT BMI was assessed for direct toxicity against L929 fibroblasts. Despite NIPAM being toxic on its own, Deng and co-authors reported otherwise [7]. In fact, after 3 days, a higher number of cells was reported for the NIPAM/LAP/CNT sample with respect to the control group. The authors did not discuss or hypothesize what could be the reason for that difference after 3 days of culture, though it could be related to the presence of LAP, which was already reported to favor cellular adhesion and proliferation. For instance, the presence of LAP in a cell-laden BI made of methylcellulose (MC) and ALG reported homogeneous cellular distribution and higher viability (70–75%) than the corresponding sample without clay (60–65% viability) in a time period of 7 days [73]. The number of living cells was stable inside the 3DBP construct with LAP (3% *w*/*w*), whereas it reduced when the nanoclay was not present. Another example of improved biocompatibility and cellular viability results related to LAP can be found in a BMI made of 0.1% *w*/*w* LAP and TEMPO BC/ALG. This formulation improved the in vitro L929 cells proliferation in the early stages. On the other hand, the cells cultured on TEMPO BC/ALG with 0.5% *w*/*w* of LAP “show better growth with larger spread area” than those on the corresponding construct with 0.1% *w*/*w* of LAP [104].

The combination of LAP, AGA and PAM was demonstrated to be non-toxic to osteoblasts [99]. More precisely, an osteoblasts co-culture viability >90% was found after 3 days of contact with the 3D printed constructs. Poly(3,4-ethylenedioxythiophene) (PEDOT) 3D printed implants intended for the restoration of neurological functions were combined with LAP and PAAM [100]. Intimate interactions between the implant and the cells are crucial for optimal regeneration performance. Moreover, the system must mimic the ECM as much as possible for neurons to make direct connections with the implant. In these terms, the cellular adhesion over the raw LAP-PAAM-PEDOT printed structures was not reported by Tondera and co-authors, leading us to think that the system by itself was not able to favor cellular adhesion. Instead, a “biomatrix” made of PEG and dextran sulfate was used to functionalize it and favor the adherence of human-induced pluripotent stem cells and neurons [100]. On the contrary, an SPE/LAP BMI intended for lower limb prosthetic socket implants showed to be not cytotoxic to the SH-SY5Y neuroblastoma cell line, demonstrating its potentiality in neural applications. These in vitro cytotoxicity evaluations were performed both by indirect and direct methods. Nevertheless, the authors also reported that some improvement should be performed since cells grew to form clusters on the hydrogel surface (lack of cellular spreading). Apart from that, improvement in some mechanical properties such as the elastic modulus could allow for higher neural viability and neurite extension of the cells [103].

Shin and co-authors [92] recently put the spotlight on the importance of the BI to closely mimic the biological cues of the native extracellular environment, which is of special interest for tissues such as the myocardium. Despite the fact that other natural and synthetic hydrogels are considered biocompatible and allow for 3DP technology, none of them are able to reproduce the properties and performance of the native ECM. The use of decellularized extracellular matrix (dcECM) in TE has several advantages since it preserves some of the growth factors, proteins and glycosaminoglycans specific to each tissue. Nonetheless, dcECM itself is not adequate for 3DBP due to the lack of mechanical and structural stability, which is further lost after decellularization. Therefore, they proposed the use of partially digested dcECM in combination with LAP and PEGDA in order to obtain a cardiac-biocompatible BI with tunable elasticity [92]. The elasticity of this kind of tissue is crucial for the proper functioning of this tissue and myofibroblast trans-differentiation. The addition of LAP to dcECM increased cellular survival after 7 days, showing the highest viability. As far as we are concerned, this is the first proposal of a BI intended for cardiac therapy in which an inorganic material is included.

**Figure 17:**
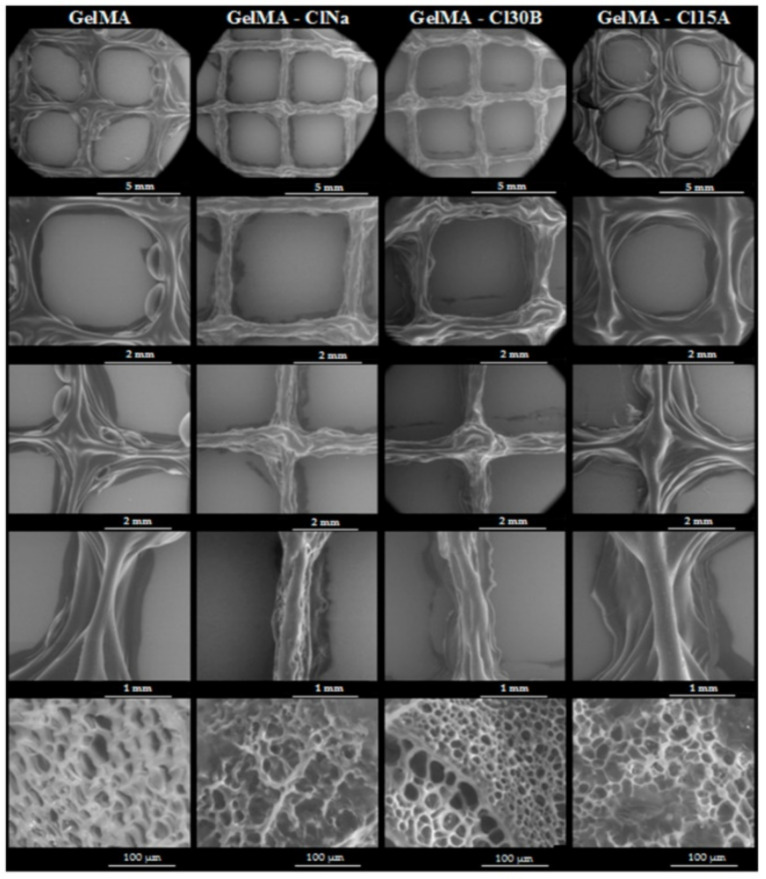
SEM microphotographs of GelMA and different Cloisite^®®^ formulations. The pore sizes and shapes vary with the type of Cloisite^®®^ used. Magnifications from top to botton: ×25, ×50, ×50, ×100 and ×1000. Reproduced with permission from [105], MDPI, 2021.

The inclusion of inorganic materials as “nanofillers” of biocompatible polymers increases the surface irregularities and porosity of the resultant scaffolds [105,120]. Particularly in the case of BI and BMI, LAP was reported to be useful to increase the porosity of GelMA and GG 3D printed constructs, and being potentially favorable for cellular growth and enabling more effective diffusion of gases and nutrients through the constructs [96,115]. Other natural layered clay minerals such as MMT also reported similar results with GelMA [105]. More precisely, the SEM analysis of GelMA printed structures combined with organo-modified and non-organo-modified MMT (Cloisite^®^ Na vs. Cloisite^®^ 15A and Cloisite^®^ 30B) reported that the porosity size and shape depend on the type of clay (Figure 17). Therefore, Cloisite^®^ Na created larger pore sizes in comparison with the pores of the constructs made of organo-modified clay minerals. On the other hand, the highest pore roundness was ascribed to Cloisite^®^ 15A and Cloisite^®^ 30B [105].

#### 2.3.2. Biodegradation of 3D Printed Constructs

Owing to the intrinsic nature of the major part of BI and BMI ingredients, bioprinted 3D constructs tend to degrade with time, especially during in vitro or in vivo conditions. The degradation rate of the 3D printed constructs is crucial to obtain a correct performance, and it should be controlled in accordance with the final scope of the printed structure. When it comes to TE, the degradation of the printed constructs will be determined by the tissue regeneration speed (the healing process of the native tissue). The different interactions between clay particles and the rest of the ingredients can interfere, under some circumstances, interfere with the degradation rate of the printed constructs. In other words, clay minerals can act as degradation rate controllers of 3D printed constructs.

The addition of 6% of LAP to 10% (*w*/*v*) PEGDA did not influence the degradation rate of the cultured 3D construct with respect to pure PEGDA [40]. On the contrary, the same concentration of LAP with 0.5% of ALG or 20% of GEL (*w*/*v*) proved useful to slow down the degradation. GEL/LAP and ALG/LAP reported approximately a 20% reduction in their degradation rates within 7 days, while pure GEL degraded in 1 day and pure ALG took 3 days.

With PEGDA being such a stable polymer, it was proposed to create recoverable enzyme-laden constructs for biocatalytic purposes [81]. For this particular scope, the resultant printed structure should retain its form in the long term in order for it to be reused and recycled. Therefore, a very slow or no degradation rate is preferred in these cases. Two-layered clay minerals were added to improve printability: HT and LAP. The PEGDA/HT structures showed faster degradation (within hours) than PEGDA/LAP, demonstrating LAP to be a better option in this particular case. Nonetheless, since HT gave better shape fidelity and resolution than LAP (Figure 11), the use of HT could be considered for other scopes such as the formulation of 3D printed modified drug delivery systems or dissolvable tailor-made supports, implants or devices. It is also worth mentioning that both ingredients (LAP and HT) exerted a minor influence on the intrinsic activity of the laden enzyme, guaranteeing the activity of the protein [81].

**Figure 18:**
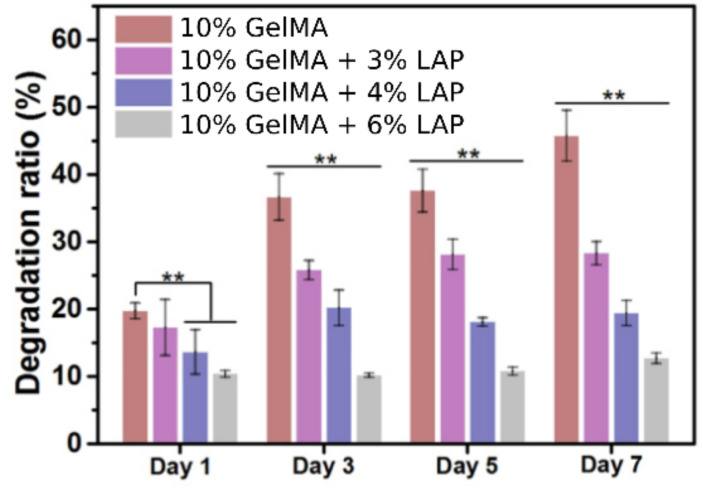
Degradation ratio of different GelMA/LAP bioinks for 7 days. Double asterisk (**) indicates significant differences between groups with *p*-value < 0.01 (ANOVA). Reproduced with permission from [96], IOP Publishing, 2019.

A BMI made of GelMA and LAP was reported to possess good biocompatibility against human umbilical cord vein endothelial cells (HUVEC) [96]. Good cellular adhesion and proliferation were also found, notwithstanding the presence of LAP in the printed scaffolds. On this particular occasion, the presence of LAP was also able to reduce the degradation rate of the printed scaffolds in a directly proportional manner to the inorganic ingredient concentration (Figure 18). In fact, an ear-printed shape remained stable after 1 week, suggesting that the scaffolds are useful for long-term TE treatments.

Lysozyme and bovine serum albumin (BSA) were chosen as analogs of bone morphogenetic protein-2 (BMP-2) and vascular endothelial growth factor (VEGF) [115]. The final scope was to study the loading capacity and the release of both molecules (lysozyme and BSA) from a BMI made of GG and LAP. Briefly, the 3DBP constructs were immersed in solutions of each molecule to study the adsorption rate; subsequently, the release was studied through collagenase digestion. The presence of LAP improved the adsorption rate for lysozyme with respect to the pure GG construct, while it did not report differences for BSA. Nonetheless, it helped to sustain the release of both molecules, even in the presence of collagenase. This interesting result indicated that the sustained release of the factors was more related to a delay in degradation of the constructs provided by LAP than to active retention of the molecules under study [115].

**Figure 19:**
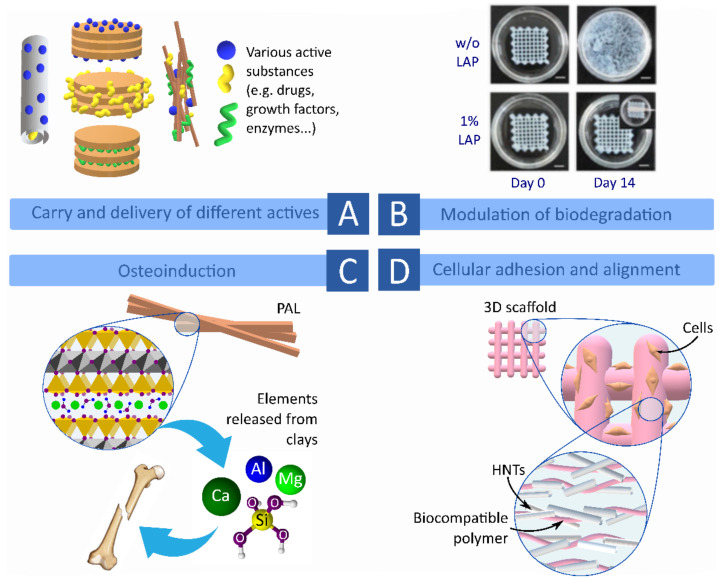
Schematic representation of some of the functional roles of clay minerals in 3DP and 3DBP constructs. (**A**) Clay minerals as carriers of different active substances. (**B**) Effect of LAP in biodegradation of ALG and TEMPO BC. Photos were reproduced with permission from [104], Elsevier, 2020. (**C**) Osteoinductive effect of clays. (**D**) Clays were also proven to improve cellular adhesion and alignment.

The addition of increasing concentrations of LAP to a BMI made of TEMPO BC and ALG [104] was demonstrated to significantly improve the structural stability of the 3D printed constructs (Figure 19B). Although no biodegradation studies were performed on this occasion (since authors were dealing with a BMI, not a BI), this result could infer the hypothesis that the in vivo degradation rate of the implanted construct could potentially be modulated by optimizing LAP concentration.

There are also studies relating the degradation rate of the 3DBP constructs with the presence of clay but in an inversely proportional way. In other words, some studies have proven that the addition of clay minerals accelerates or favors the degradation of the printed constructs. For instance, the degradation rate of GEL/ALG/LAP printed constructs was also related to the concentration of LAP particles within the printed structure [98]. This degradation was monitored in buffer solution at 37 °C for 14 days. In comparison with the constructs without LAP, those with 1% nanoclay had a more controlled gelatin release behavior within 14 days of incubation, followed by a decrease in the release rate after 2 weeks. Nonetheless, all the printed structures with LAP in their composition reported a faster degradation than the GEL/ALG counterpart (without LAP). Authors ascribed this construct “liability” to the presence of LAP agglomerates due to improper clay dispersion within the system. In a more recent study, the natural layered clay mineral MMT was combined with GelMA [105]. Two organo-modified MMT (Cloisite^®^ 30B and Cloisite^®^ 15A) and a non-functionalized MMT (Na Cloisite^®^) reported differential degradation rates of the GelMA printed constructs. First of all, in comparison with pure GelMA, the addition of MMT emphasized the degradation of the resultant hydrogels, something in agreement with the study of Cai and co-workers [98]. In the study performed by Alexa and co-workers, they stated that Cloisite^®^ 30B and Cloisite^®^ 15A were more difficult to disperse homogeneously within the GelMA hydrogel; this is why they frequently clogged the printing nozzle. Nevertheless, the functionalized clay (Cloisite^®^ 30B, which was not properly dispersed according to authors) reported the lowest degradation rate, followed by pure GelMA and GelMA/Na Cloisite^®^ (more properly dispersed) [105]. These results partially confirmed the hypothesis of Cai and co-authors [98] but also raised new doubts about the mechanism of action through which layered clay minerals accelerate the degradation of gelatine-based 3D printed scaffolds. In any case, the homogeneous dispersion of clay particles within the formulation is of great importance not only for proper printability (nozzle clogging, etcetera) but also for long-term stability. This homogeneity should be accomplished by means of a proper preparation procedure of the BI or BMI (pre-printing stage).

#### 2.3.3. Carriers and Control Release of Functional Ingredients

Owing to the great adsorption capacity of clay minerals, they were widely explored as potential drug delivery systems of conventional and modified dosage forms (Figure 19A) [121]. Equally, in 3DBP, it is possible to find different studies dealing with the influence of inorganic ingredients in the controlled release of bioactive substances. VEFG and BSA are some of the most frequently explored. The combination of clay minerals with traditional drugs usually leads to an irreversible release of a certain drug fraction that remains “adsorbed” by the phyllosilicate [61,122]. This phenomenon was also reported for VEGF and BSA molecules included within 3D constructs. It is particularly important to explore the release profile and to optimize the concentration of both the active ingredient and the clay mineral, to ensure the release of a proper and effective dose, something that must be performed on a case-by-case basis. Nevertheless, unlike oral clay-based dosage forms (which have limited time to release the active ingredient), the 3D bioprinted constructs will presumably remain in the targeted tissue for longer times (>8–10 h), and most of them are designed to be completely degraded.

**Figure 20:**
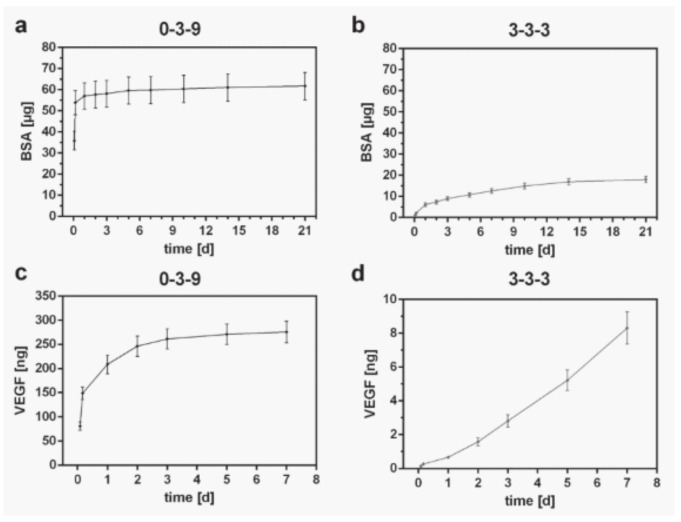
Release of BSA and VEGF proteins from MC/ALG/LAP constructs. The 0–3–9 stands for constructs made of 0% LAP, 3% ALG and 9% MC (wt), while 3–3–3 stands for 3% wt concentration of all the ingredients. Cumulative release curves of BSA (**a**,**b**) and VEGF (**c**,**d**). Reproduced with permission from [73], Elsevier, 2017.

The release of BSA and VEGF proteins from an MC/ALG/LAP BI was strongly influenced by the presence of the nanoclay (Figure 20) [73]. Both proteins suffered from burst release in the absence of LAP, reaching a plateau within 1 or 2 days for BSA and 3 days in the case of VEGF. When the nanoclay was present, sustained BSA and VEGF releases were found. The authors stated that significant amounts of growth factors were retained by the clay since none of them was completely released in 21 days [73]. Even if the total dose of VEGF and BSA was not released within 21 days, that does not imply that LAP would completely retain the rest of their doses. Unlike BSA, which showed a progressive reduction in the release rate of the protein (reaching a plateau in 14 days), the VEGF release profile was almost linear during the whole experiment (21 days). These results indicate that longer times are needed for the growth factors to be released. In fact, during the whole period tested, the cumulative release of BSA and VEGF continued to increase. A reduction in the final porosity of the construct in the presence of LAP together with the possible electrostatic interactions between LAP, VEGF, BSA and ALG were identified by authors as the responsible factors of the sustained slow release [73]. The influence of LAP in the VEGF release was also addressed by Peak and co-workers. In this case, fibroblast growth factor (FGF) was also explored, and both molecules were released from a 3D bioprinted construct made of PEGDTT and LAP [82]. A 20% VEGF and FGF were released after 7 days, reaching completion after 28 days. The rapid migration of HUVEC cells was ascribed, indeed, to the sustained release of these actives from the printed structure. The release profile obtained was ascribed to both the adsorptive capacity of LAP and to the degradation rate of the system, which was also controlled by the clay nanoparticles (due to buffering activity of LAP). The degradation rate of PEGDDT was found to be significantly shorter in water than in PBS solution due to the pH modification caused by LAP. “Equilibrium pH of nanosilicates (i.e., pH 9.4) causes a decrease in time needed for PEGDTT samples to degrade” [82].

Apart from a controlled release of active substances, the presence of clay minerals such as LAP was also shown to be useful to rapidly retain significant amounts of growth factors within the bioprinted constructs. In this sense, Cidonio and co-workers demonstrated that the presence of just 1% (*w*/*v*) of LAP was enough to obtain very effective adsorption of lysozyme and BSA [115]. The effective role of LAP as a release controller of growth factor was proved by Cidonio and co-workers in a more recent study, where the LAP-based BI promoted vascular ingrowth and bone tissue regeneration in vitro, ex vivo and in vivo [114]. Therefore, BSA release can be effectively controlled and sustained with time by the addition of 0.5% *w*/*w* LAP to TEMPO BC and ALG BMI [104]. In particular, this study revealed that the addition of 1% *w*/*w* of LAP in the BI proved to be excessive, with practically no BSA release. Similar results were reported with a wide variety of other molecules such as drugs [121].

**Figure 21:**
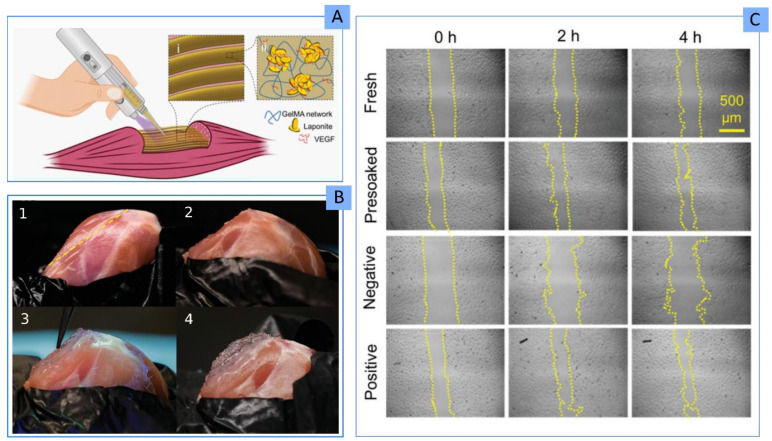
(**A**) In vivo handheld printing device aspect and composition of GelMA-LAP BMI, loaded with VEGF. The aim of this BI and the handheld device was to treat skeletal muscle injuries through in situ 3D printing. (**B**) Photographs of muscle-induced injury. Step 3 shows the handheld printing process, and Step 4 shows the finished restoration surgery. (**C**) Optical microphotographs of scratch assay with HUVEC cells. “Fresh” results belonged to GelMA-LAP/VEGF BMI crosslinked and subjected to scratch assay at time 0; “Presoaked” scratch assay was performed with crosslinked GelMA-LAP/VEGF BMI pre-soaked in DPBS for 24 h before scratch assay; “Negative” control was composed of growth medium without VEGF; “Positive” control test was performed by means of growth medium supplemented with 30 ng/mL of VEGF. Figures adapted with permission from [63], IOP Publishing, 2021.

Direct in situ 3DBP processes are an advanced approach for TE and regenerative medicine. These processes attempt to grant the practitioner the ability to print in situ tailor-made 3D constructs perfectly adapted to the targeted area through a handheld device. In these cases, the shape fidelity cannot be measured since there would be no “digital design” to compare the construct with. Nevertheless, printability is still of great importance. Thanks to the addition of LAP to GelMA polymer, Quint and co-workers were able to formulate a supportive and adhesive BMI for in vivo handheld 3DBP with in situ photo-crosslinking (Figure 21) intended to stimulate muscle regeneration [63]. On this occasion, the attention was centered on the role of LAP as a VEGF carrier and release platform. This growth factor was loaded into LAP through electrostatic interactions, and then the LAP-VEGF nanocomposite was subsequently incorporated into GelMA hydrogel. A steady release of VEGF over a 3 weeks period was observed, though there was a significant proportion of non-released growth factors. Despite that, the scratch assay demonstrated effective HUVEC cells stimulation [63].

#### 2.3.4. Clay Minerals as Functional Ingredients of Bioinks and Biomaterial Inks

Several activities were associated with clay minerals, thus acting as active substances themselves. Their activities as gastrointestinal protectors, laxatives and antidiarrheals are widely known [123]. Their favorable roles during wound healing were also extensively reviewed, acting as hemostatics, wound healing promoters, antimicrobial agents and useful wound dressing ingredients [15,42]. Apart from that, Gaharwar and co-workers reviewed some immunomodulatory activity of clay minerals ascribed to their chemical composition. More precisely, Li^+^, Mg^2+^ and Si(OH)_4_ were proven to enhance or trigger immunological activity. Even if this statement would be contradictory to the desirable properties of a BI or BMI, some studies reported a favorable tissue regeneration induced by these immune responses [124,125].

Despite these already known features of phyllosilicates, in the specific field of 3DBP, the usefulness of clay minerals’ therapeutic activities is still an unexplored topic. The main results so far deal with their activities as osteoinductive agents. According to a recent scientometric analysis, the most frequently reported bioprinted tissues within the last two decades are cartilage and bone [1]. Bone tissue regeneration is based on the synthesis of proper scaffolds able to facilitate cellular growth and to maintain the functional tissue construct until the total reestablishment of the damaged area. However, it is still a great challenge to achieve a proper level of cell deposition and distribution in 3D bone scaffolds together with adequate mechanical properties.

Clay minerals reported outstanding results in bone and cartilage tissue regeneration in vitro [126] since both natural [127] and synthetic clay minerals [116,128,129] have the ability to stimulate bone regeneration thanks to their chemical composition (Figure 19C). The exact mechanism of the action exerted by clay minerals in osteoinduction was related to the release of elements from the clay structure [85,89], the triggering of certain immunomodulatory responses [130] and the cellular endocytosis of clay particles [116]. In the latter case, transcriptome monitoring of human mesenchymal stem cells (hMSC) subjected to direct contact with LAP particles revealed that more than 4000 genes were differentially expressed due to the presence of the clay mineral. Carrow and co-workers stated that “nanosilicate attachment to the cell membrane and subsequent cellular internalization activated stress-responsive pathways such as mitogen-activated protein kinase (MAPK), which subsequently directed hMSC differentiation toward osteogenic and chondrogenic lineages”.

N-acryloyl glycinamid (NAGA) polymer-based BI combined with LAP BI was evaluated in vitro and in vivo for biocompatibility and osteoinductive activity [89]. Firstly, an increase in the concentration of NAGA was demonstrated to hinder cellular viability, something that was ascribed to an excess of compactness of the BI. The presence of 7% *w*/*v* LAP did not interfere with the viability of primary rat osteoblasts. Moreover, LAP was considered responsible for the release of Si and Mg cations, which were able to diffuse throughout the construct. Nonetheless, the increase in NAGA concentration (with constant LAP concentration) reduced the release rate of these ions. The interference of NAGA with Si and Mg release was ascribed to the reduced porosity, which could be a factor to bear in mind for controlled release experiences. The activity of Si and Mg in osteogenic differentiation was confirmed by the in vivo studies, where the NAGA/LAP implanted constructs reported new bone formation. In comparison with the control, the NAGA/LAP 3D printed construct produced thicker tissue formation. Moreover, no inflammation or other immune responses were detected [89]. The osteoinductive activity of LAP was used later by the same research group. On this occasion, a PEGDA/LAP BMI was combined in situ with a cell-laden HA BI [71]. Both parts were combined during the 3DBP process by using the so-called “two-channel method”, in which the HA BI provided the osteoblasts and protected the cells from the UV light applied during the post-processing step, whereas the PEG-LAP BMI acted as a supporting construct that provided stability, oxygen and nutrients (including Si and Mg) toward the cells. In fact, tibia repair and ectopic osteoinduction experiments confirmed that the environment around PEG-LAP favored cellular differentiation [71]. Similarly, Byambaa and co-workers reported an osteoinductive activity of Na^+^, Mg^2+^, Si(OH)_4_ and Li^+^ released by LAP [85]. Even if the release of these elements was not monitored in this study, the mineralization was found to be higher as higher the LAP concentration (10–100 µg/mL), which allowed to confirm the active participation of the clay mineral [85]. Likewise, the osteogenic cellular differentiation effect was also observed in GG/LAP constructs, as proved by an increased expression of alkaline phosphatase [115]. LAP dose-dependent bone mineralization was also supported by a BI formulated with MC/ALG/LAP, together with VEGF and BMP-2. The resultant 3DBP constructs were confirmed as viable and functional bone implants by means of in vitro, ex vivo and in vivo studies [114]. Together with LAP, hydroxyapatite was also shown to be a useful inorganic ingredient to carry and control the release of VEGF and BMP-2 growth factors from an MC/ALG BI [131]. This controlled and sustained release reduced the necessity of using supraphysiological doses of growth factors, thus reducing undesirable side effects.

**Figure 22:**
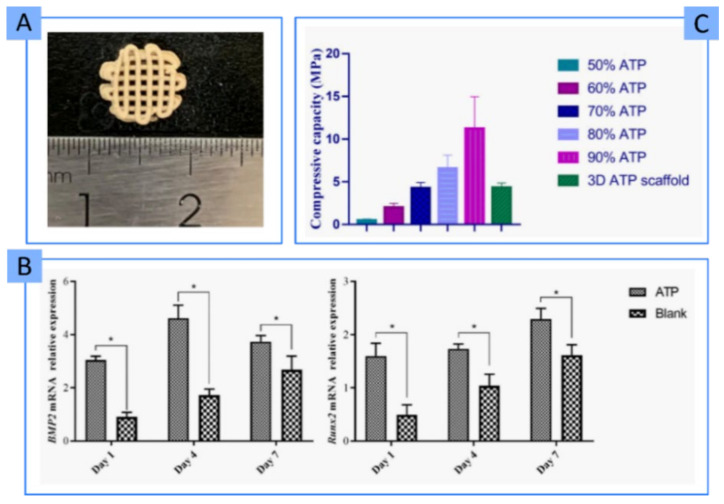
(**A**) Photograph of the PAL 3D printed scaffold. (**B**) mRNA expression of BMP2 and Runx2 genes quantified by real-time RT-PCR (*p*-value < 0.05). ATP stands for “PAL” and Blank stands for “control group” indicating that those rats did not receive any treatment. (**C**) Compressive strength of PAL scaffolds at different concentrations of clay minerals. Figures adapted from [90], Dove Medical Pres Ltd, 2020.

The osteoinductive activity of natural clay minerals was also shown. Indeed, a natural PAL was used as the main and major ingredient of 3D printed bone regeneration scaffolds in combination with polyvinyl alcohol (PVA) as a binder (Figure 22A) [90]. Due to the natural origin of the PAL sample (not commercial), it would have been convenient to perform an assessment of the sample richness in order to discern the possible influence of secondary mineral phases present (such as carbonates or hydroxyapatite). Nonetheless, no information was provided on this matter, though the authors reported that a PAL purification procedure was performed. In vitro (human Bone Marrow Stromal Cells, h-BMSC cells) and in vivo (rat) biocompatibility and osteogenesis tests were performed. Besides total biocompatibility, the PAL scaffolds induced direct bone formation by membrane ossification and re-vascularization in vivo. This effect was ascribed to the induction of BMP-2 and Runx2 genes, detected through real-time RT-PCR (Figure 22). Since the tested scaffold was purely made of PAL (the PVA was eliminated through carbonization at 500 °C before in vivo implantation), the osteoinduction was strictly ascribed to the inorganic ingredient.

### 2.4. Mechanical Reinforcement of Bioinks and Biomaterial Inks

Together with rheological modifications, another strong suit of clay minerals is their role as mechanical reinforcement of a wide variety of ingredients. In particular, they pose strong constraints on mechanical strength [132,133]. One of the main disadvantages of TE and 3DBP constructs is their low mechanical resistance, mainly due to the nature of the main ingredients (natural and/or synthetic hydrogels), the high amount of water and their biodegradability. Despite the widely accepted usefulness of clay minerals as polymeric reinforcement (especially of the organo-modified ones), the 3DBP is a novel and quite recent technique that introduces new factors to bear in mind. Consequently, newly, specific studies dealing with BI and BMI mechanical properties are needed. The existing literature reveals that the mechanical performance of clay minerals depends on the interactions between clay particles, clay particles and the rest of the BMI or BI ingredients, as well as the possible clay–cells interactions. The complexity of the BI and BMI formulations challenges the optimization of the mechanical resistance provided by clays. In this scenario, the purity and richness of the inorganic ingredient are of great importance to maximize the final performance and minimize the influence of external, unpredictable factors. This may be an explanation for the number of studies dealing with synthetic clay LAP with respect to natural ones, which are usually associated with a significantly higher number of impurities.

Young’s modulus and energy dissipation of PEGDA, GEL and ALG were significantly increased by the addition of 6% *w*/*v* of LAP [40]. This result was associated with the high surface interactions between LAP layered nanoparticles and the polymers. Moreover, the physical interactions between the nanoclay and the polymer chains award the final formulation with viscoelastic properties, which could explain the energy dissipation.

**Figure 23:**
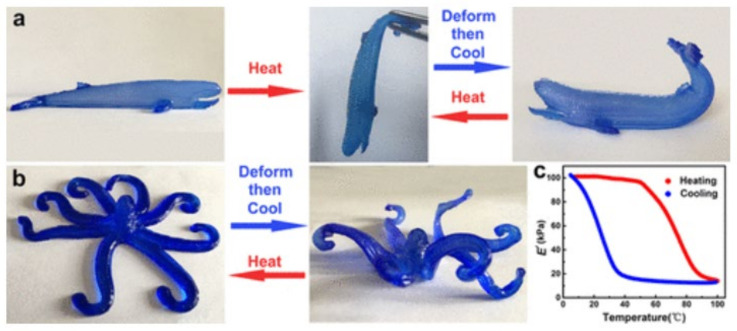
Shape modification of the 3D printed structure due to sol–gel transformation of AGA. The softening and hardening cycles of complex printed structures (**a**,**b**); changes in storage modulus of the hydrogels during heating/cooling cycles; (**c**) variations of storage modulus during heating and cooling cycles. Reproduced with permission from [99], American Chemical Society, 2018 .

The addition of LAP to an AGA/PAM nanocomposite provided better tensile and compression strength [99]. Moreover, this hydrogel was demonstrated to be “recoverable” in terms of shape and consistency. That is, the damage exerted over the constructs after the mechanical tests can be “healed” by heating the construct and “remodeling” its shape (thanks to AGA) while maintaining its mechanical properties (due to LAP), Figure 23.

LAP also proved to improve the mechanical properties of GelMA constructs. A printed grid scaffold with 14 layers using GelMA/LAP (10% of GelMA and 4% or LAP) showed to maintain either the grid pores of the digital design and also lateral pores formed during the layer deposition, which is something not happening for those constructs with lower or no LAP concentration [96]. This result was related to the higher mechanical support that LAP gave to the hydrogel, thus allowing pore integrity. The strength and compression modulus of this GelMA/LAP BMI also increased with increasing LAP concentration, with the failure strain values not being affected by the clay presence [96]. Likewise, there are studies reporting that the addition of clay does not have a significant effect on the mechanical properties of GelMA BI or BMI. A BMI made of GelMA and LAP withstood compression at 50% strain and reached up to 4.5 kPa as maximum compressive stress [63]. This value in comparison with native murine skeletal muscles (experiencing a maximum of 40% of strain during contraction) demonstrates that GelMA/LAP BMI will allow the printed constructs to deform during movement. Nonetheless, the presence of LAP did not exert significant changes in the mechanical properties with respect to the corresponding counterpart without LAP. Bearing in mind some factors already discussed in this review, we hypothesize that this ineffective influence of LAP over mechanical properties could be potentially related to inadequate clay concentration (not specified by Quint and co-workers) or improper clay dispersion (vortex mixer was used for only 1 min) [63].

Another strategy with which clay minerals were reported to increase the mechanical performance of natural hydrogels is the use of the two-channel method [71]. In this study, LAP was combined with PEGDA and acted as a supportive BMI of a cell-laden HA BI. This idea consists of using a more viscous BMI intercalated between the BI layers. In particular, the compression strength values and compression modulus increased 4–3 times after the addition of 7% *w*/*v* of LAP to PEGDA. It is also worth mentioning that the Transmission Electron Microscopy (TEM) study demonstrated a homogeneous dispersion of LAP nanoparticles within the BMI [71], proving that a homogeneous clay distribution leads to better results. The mechanical performance of a BMI made of AGA, GEL and LAP was monitored over time to reveal the influence of the in vitro conditions on the scaffold maturation. The compressive modulus of the 3D printed constructs without LAP decreased from day 0 to day 14. Contrary to expectations, the addition of LAP at 0.5 and 1% further reduced the compressive modulus, even at time 0. This worsening of the BMI compressive modulus after the addition of LAP was ascribed to the presence of LAP agglomerates within the hydrogel network due to improper or poor clay particles dispersion [98].

The tensile strength, Young’s modulus, elongation at break and compressive stress of a NAGA polymer-based BI were improved by the “dual amide hydrogen bonding interaction and physical crosslinking of the nanoclay–polymer chain” [89]. In fact, the constructs with LAP were able to withstand strain, stretching and compression without damage. Additionally, the swelling maintained stable and unchanged for longer times with respect to the control, which is of great interest in reproducibility and maintenance of the mechanical properties.

The thermoresponsive monomer NIPAM was combined with CNT and LAP to prepare a BMI [7]. In this BMI, LAP was used as a physical crosslinker of the whole system. All the BMIs prepared presented remarkable mechanical properties, which were clearly improved by the presence of LAP in a directly proportional manner: as higher the LAP concentration, the higher the compression modulus, the elongation at break and the tensile strength. Just as an example, the tensile stress improved from 0.18 MPa for the BMI with approximately 7% *w*/*w* of LAP to 0.53 MPa, with 22% *w*/*w* of clay mineral (3 times higher). The same increasing order was found for the compression modulus, whereas the elongation at break was less affected by LAP, although increased values were also reported [7].

PEDOT was used as a base ingredient for the fabrication of 3D printed neuroprosthetic implants to restore neurological functions [100]. Unfortunately, PEDOT does not possess adequate mechanical and conductive properties for this scope. For instance, the printed structures must resist deformation during body movements, handling and implantation. Tondera and co-workers proposed the use of LAP and PAAM to provide elasticity, mechanical resistance and improved conductivity to the formulation. With respect to tensile tests, the printed construct including LAP withstood 800% tensile strain before breaking. In addition, it showed plastic deformation and recovery of the original length upon strain relaxation. Regarding the conductivity, PEDOT typically requires to be doped with an anionic polyelectrolyte such as polystyrene sulfonate to ameliorate its conductivity and dispersion in water. The remarkable conductivity of the resultant LAP/PAAM/PEDOT system was ascribed to the “introduction of mobile charge carriers (holes) in the PEDOT backbone compensated by negative charges on the surface of LAP crystals” [100]. This means that LAP, apart from playing a crucial role in the final mechanical and rheological properties, was also providing the system with good electrochemical stability.

In the same manner, as the concentration of clay influences the final printability, an optimal concentration of clay is necessary to obtain good mechanical properties. The addition of small amounts of LAP (0.1% *w*/*v*) to a TEMPO BC/ALG hydrogel improved the compression strength of the bioprinted structure from approximately 250 kPa to 330 kPa, while higher LAP concentrations had little or no effect on the compressive strength [104]. The participation of LAP in the crosslinking process of TEMPO BC and ALG fibers could explain this result since the “active” sites with which LAP could interact during the crosslinking could be exhausted at 0.1% *w*/*v*, thus higher LAP concentration not having a significant effect on the mechanical properties.

Despite the purity and richness provided by LAP, natural clay minerals are accessible, abundant, sustainable and affordable raw materials. After proper purification, they can also exert outstanding mechanical reinforcement for BI and BMI. For instance, the usefulness of natural MMT as a mechanical reinforcing agent was reported in combination with GelMA [105]. In particular, three types of natural MMT, commercially available, were combined with GelMA: sodium Cloisite^®^ (natural MMT, Na Cloisite^®^), Cloisite^®^ 30B and Cloisite^®^ 15A (functionalized MMT). The storage modulus indicated an increase in the mechanical stability and stronger gel structure when MMT was added. Under stress conditions, the addition of MMT helped to dissipate the compressive energy, notwithstanding the type of clay. Nevertheless, the combination of GelMA with functionalized clays (Cloisite^®^ 30B and Cloisite^®^ 15A) showed to be stronger than GelMA/Cloisite^®^ Na [105], in agreement with the results found by Paspali and co-workers [25].

**Figure 24:**
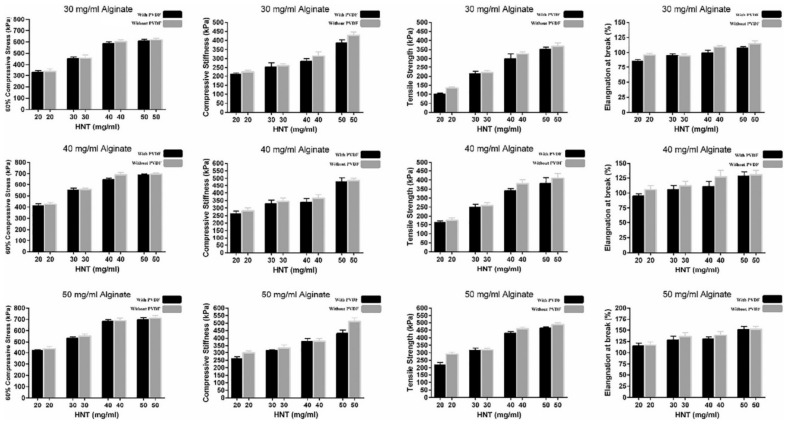
Compressive modulus, compressive stiffness, tensile strength and elongation at break of biomaterials combined with different concentrations of HNTs. Reproduced with permission from [91], Elsevier, 2018.

The natural, tubular clay mineral HNT was also tested as mechanical reinforcement in 3DBP. In particular, HNTs were proposed as BMI ingredients in a study dealing with cartilage regeneration [91]. Different concentrations of ALG, MC, polyvinylidene fluoride (PVDF) and HNTs were combined to create a battery of BMIs that were subsequently subjected to 3DBP. Both the tensile strength and compressive stress increased with HNTs (Figure 24), so this natural clay was also identified as a good alternative to reinforce polymers.

High amounts of purified PAL (50–90% *w*/*w*) were combined with 10% of PVA and extruded to produce ≈1 mm scaffolds intended for bone tissue regeneration [90]. No cells were laden on this BMI since, after the 3DP, the constructs were dehydrated and sintered at 500 °C to carbonize PVA. Mechanical tests showed that the compressive capacity increased with the amount of PAL (Figure 22C). Therefore, the formulation with 90% of PAL was selected and further characterized. In particular, the produced scaffold reported a longitudinal compression strength of 4.3 MPa, very similar to the compressive strength of native cancellous bone [90].

## 3. Future Prospects of Clay Minerals in 3D Bioprinting

At this point of the review, it is clear that one of the strong suits of clay minerals is their rheological properties, which were exploited in a wide variety of areas such as building industry, ceramics, drilling fluids, pharmaceutics, etc. [17,19,134,135,136,137,138]. Moreover, both synthetic and natural clay minerals have proven to play a crucial role as mechanical enhancers and functional ingredients. They even demonstrated extensive use as functional ingredients. Therefore, it is plain to see the potential of these minerals in 3DBP and TE, which has not been sufficiently exploited yet, probably due to its recent emergence. The combination of an emerging technique as versatile and advantageous as 3DBP together with the variety, abundance and versatility of clay minerals makes this a profitable field of study with a wide variety of possibilities and opportunities. This section is dedicated to the potential, unexplored or uncharted uses of clay minerals in 3DPB and TE.

**Figure 25:**
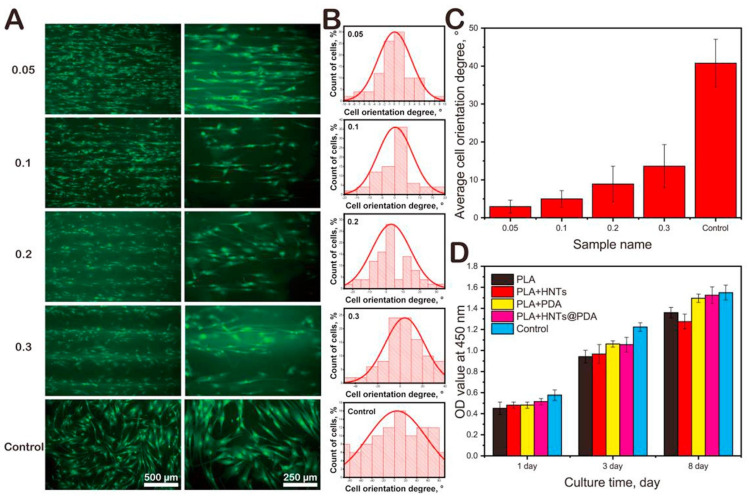
(**A**) hMSC seeded over PLA/HNT 3D printed scaffolds and stained with fluorescein isothiocyanate. Authors reported different stripe widths. (**B**) Cell counting and orientation degree; the average cell orientation degree was summarized in (**C**). (**D**) Results of cell proliferation (CCK-8) for 1, 3 and 8 days. OD stands for “optical density” quantified at 450 nm. Reproduced with permission from [140], Elsevier, 2019.

Until now, all the studies mentioning improved cellular adhesion and 3DBP were dealing with LAP (synthetic clay). Nevertheless, the promotion of cellular adhesion is also a well-known feature of natural clay minerals [43,59,139]. As an example, Naumenko and co-workers proposed an implantable scaffold made of chitosan (CS), AGA, GEL and HNTs for TE [120]. In this particular study, no cells were added to the scaffold, and no 3DP nor 3DBP technology was used. Nonetheless, results revealed that the scaffold was biocompatible (both in vivo and in vitro), and, more importantly, cells were tightly attached to its surface (Figure 19D). The in vivo studies in Wistar rats also showed full restoration of blood supply in 6 weeks [120]. Moreover, the hydrophilicity and cellular adhesion of 3D printed structures of PLA were improved by HNT coating [140]. Therefore, the clay mineral was not taking part in the BMI formulation (and therefore, not taking part in the 3DBP process). This approach was based on the idea that certain PLA printed patterns were able to guide cellular orientation by means of “contact guidance” (Figure 25). Cellular orientation is of use in the fabrication of cell-based biosensors, neural chips, cell arrays or wound healing. Nonetheless, PLA does not show enough cellular adhesion for this hypothesis to be proved. Thus, they turned to HNTs to improve cell affinity over PLA structures and ultimately guide the orientation of stem cells.

The remarkable importance of clay minerals in drug delivery could also allow for the carrying and controlled release of different drugs and bioactive molecules (such as growth factors) in the targeted tissue. In this regard, LAP was already mentioned as a carrier of VEGF and BSA in some BMI and BI [63,73,104,115]. Regarding other clay minerals, HNTs were proposed as carriers of Ag in a 3D printed PLA construct intended as a biomedical device to be used in wound healing [24]. This study was not included in any of the previous sections because HNTs were not a strict part of the BMI. Precisely, the PLA constructs (already printed) were subsequently soaked firstly into NaOH (to increase hydrophilicity) and then in a CS-ZnHNT-Ag suspension. Therefore, HNT was used as a post-3D printing functionalization ingredient to carry and release Zn and Ag, thus providing the printed medical device with “anti-biofouling activity” against *S. aureus* and *S. epidermidis*.

The high specific surface area of clay minerals is also an attractive feature to be exploited in 3DBP and TE, especially for treatments involving adsorption mechanisms. This feature was already exploited in a wide variety of areas, from environmental remediation to healthcare and cosmetics. In fact, it is possible to find a recent study dealing with 3D printed constructs made of ALG, MMT (Cloisite^®^ 20A) and acrylic acid [141]. In this case, the aim was not to produce BI or 3D structures to be applied as implants, medical devices, or any sort, but to obtain adsorbent constructs for water remediation. The effective and non-selective metal ion adsorption capacity was found, thus indicating that the formulation was apt to be used for wastewater remediation [141]. As far as medical uses are concerned, it was also reviewed that inorganic ingredients could be able to adsorb wound debris, microorganisms and toxins from the wounded area [42]. In this regard, it is possible to hypothesize that 3D printed scaffolds with clay minerals would be potential formulations to control and/or prevent wound infections. Moreover, they are able to award wound dressings with desirable features such as proper mechanical resistance and adequate water-vapor transmission rate.

Another useful property of clay minerals is their ability to block UV radiation through a physical mechanism; this is why they are used as sunscreen actives [142,143,144]. This property can be useful to protect UV-sensitive molecules or actives within BMI or BI. In particular, acrylonitrile butadiene styrene (ABS) is a widely used industrial polymer sensitive to UV radiation, which accelerates its degradation. In a 3DP study, ABS constructs were coated with an organo-modified MMT dispersed in dimethyl ketone after the printing process. Briefly, the ABS printed constructs were immersed in different MMT suspensions for different times, and the resultant surface properties were evaluated [145]. In particular, the nanoclay coated constructs showed a significant increase in UV absorbance. Moreover, the hardness of the construct was increased (by 9.7%), and the surface roughness was reduced (by 94.9%). In this regard, we hypothesize that clay minerals could be of great use for the production of 3D printed constructs carrying UV-sensitive actives, especially if they are intended to treat skin affections and wounds (exposed to UV radiation). The introduction of clay minerals within the BI, BMI or the production of coaxial 3DP with clay minerals located in the most external layer would protect other UV sensitive molecules.

## 4. Conclusions

The adaptation and progress of the 3DP technology toward 3DBP (3D printing adapted to biomedical purposes) has opened the door to a world of new opportunities and possibilities in the biomedical field. In fact, 3DBP enables the preparation of new dosage forms, tailor-made devices and implants, tailor-made scaffolds for TE and many other individualized therapy options. Until the appearance of this technology, TE approaches were limited to two-dimensional cell sheets. Currently, 3DBP allows for the fabrication of cell-laden artificial 3D tissues that can be even shaped as desired and individualized for each patient and tissue. Nonetheless, the currently available materials used for 3DBP usually fail to provide adequate rheological and mechanical properties. In an attempt to meet these needs, synthetic organic macromolecules and inorganic materials are in the spotlight. In particular, this document has reviewed the most recent advances and reported uses of synthetic and natural inorganic clay minerals in 3DBP. The present review has allowed us to conclude the following:

- Functionalized or organo-modified layered phyllosilicates (montmorillonite or bentonite) are more prone to intercalate macromolecules within the interlayer space. This ability was proven to enhance the mechanical properties of macromolecules such as polymers;
- Fibrous (sepiolite and palygorskite) and tubular clay minerals (halloysite nanotubes) were also proven to enhance the mechanical properties of certain macromolecules by adjusting their orientation within the inkjet;
- Due to the chemical composition of clay minerals (aluminosilicates), they are promising materials for bone TE, with being able not only to provide mechanical resistance but also to trigger osteoinduction;
- Clay minerals were proven to induce cellular attachment, which is of great interest to add anchor points for cells when working with 3D constructs made of synthetic materials, for which cells usually do not show too much affinity;
- The high surface area of clay minerals makes them able to adsorb and carry a wide range of molecules. In fact, this feature was exploited throughout the years for the design and development of drug delivery systems. In the field of study at hand, clay minerals were proven to control the diffusion of growth factors within 3D constructs, something that could also be extended to the controlled release of other actives;
- Even if clay minerals are able to improve the viscosity of aqueous formulations, it is not possible to predict the final rheological behavior of the formulation. Rheological properties not only depend on the clay type and concentration but also on the preparation procedure and the different interactions between clay particles and the rest of the ingredients in the environment. Bearing in mind that BMIs and BI are usually complex mixtures, it is imperative to find the proper clay concentration for each formulation.

Finally, other uncharted or yet unexplored uses of clay minerals in 3DBP were also hypothesized based on the already existing literature. In this sense, clay minerals can also provide UV protection to UV-sensitive actives included in 3D constructs. This is of special interest for the regeneration and treatment of light-exposed areas such as facial skin. The high specific surface area and adsorption capacity of these minerals make them promising ingredients for constructs intended to control and remove unwanted substances such as debris, bacteria, fluids, toxins, etc., thus being interesting ingredients for the treatment and regeneration of wounds.

## Figures and Tables

**Table 1:** Main differences between 3D bioprinting (3DBP) and 3D printing (3DP).

Properties	3DBP	3DP
Nature, type and characteristics of the ink ingredients	Biological and biocompatible materials (BMI), sometimes laden with human or mammalian cells (BI). Liquid or semisolid, gel-like materials (aqueous-rich). Usually, post-processing steps are needed to improve the resistance and manageability of the construct (chemical or physical crosslinking methods such as light-based crosslinking, among many others).	Molten plastics, synthetic polymers, metal alloys, ceramics, concrete, etc. Solid, semisolid, biocompatible or non-biocompatible materials. Minimal/absent post-processing.
Printer features	The “bioprinters” require less robustness: they usually work at low temperatures, pressures, speeds, etc. These mild working conditions guarantee cellular viability. High precision is mandatory to reproduce native tissue structures.	Robust equipment, able to work in extreme conditions (high temperatures and/or pressures). No need to ensure cellular viability. Precision depends on the item and its final scope (i.e., less precision for building industry; higher for microchips or microfluidics).
Most frequently used techniques	Extrusion-based bioprinting, droplet (or inkjet) bioprinting and laser-based bioprinting.	Fused-deposition modelling, selective laser sintering, stereolithography, multi-jet fusion.

## Data Availability

Not applicable.

## Abbreviations

3DBP	3D bioprinting, bioprinting
3DP	3D printing
AGA	Agarose
ALG	Alginate
BI	Bioink
BMI	Biomaterial ink
BSA	Bovine serum albumin
CMC	Carboxymethylcellulose
CNT	Carbon nanotubes
CS	Chitosan
dcECM	De-cellularized cardiac extracellular matrix
dECM	De-cellularized extracellular matrix
GelMA	Gelatin methacryloyl
GG	Gellan gum
HA	Hyaluronic acid
HDPE	High-density polyethylene
HDPE	High-density polyethylene
HEK	Human embryonic kidney cells
HEMA	2-hydroxyethyl methacrylate
HNTs	Halloysite nanotubes
HT	Hectorite, Bentone^®^ MA
k-CA	kappa-carrageenan
LAP	Laponite
MC	Methylcellulose
MMT	Montmorillonite, bentonite, Veegum^®^ HS
NAGA	N-acryloyl glycinamid
NIPAM	N-isopropyl acrylamide
PAAM	Polyacrylamide
PAL	Palygorskite or attapulgite
PEDOT	Poly(3,4-ethylenedioxythiophene)
PEG	Poly-ethylene glycol
PEGDA	Poly(ethylene glycol) di-acrylate
PEGDTT	poly(ethylene glycol)-dithiothreitol
PETG	Polyethylene glycol terephthalate
PLA	Polylactic acid
PMet-b-POxa	Poly(2-methyl-2-oxazoline)-b-poly(2-n-propyl-2-oxazine
PVA	Polyvinyl alcohol
PVDF	Polyvinylidene fluoride
SEM	Scanning Electron Microscopy
SEP	Sepiolite
SF	Silk fibroin
SPE	N-(3-Sulfopropyl)-N-methacroyloxyethyl-N,N-dimethylammonium betaine
TE	Tissue engineering
TEMPO BC	2,2,6,6-tetramethyl-piperidinyl-1-oxyl-oxidized bacterial cellulose
